# Decoding reactive structures in dilute alloy catalysts

**DOI:** 10.1038/s41467-022-28366-w

**Published:** 2022-02-11

**Authors:** Nicholas Marcella, Jin Soo Lim, Anna M. Płonka, George Yan, Cameron J. Owen, Jessi E. S. van der Hoeven, Alexandre C. Foucher, Hio Tong Ngan, Steven B. Torrisi, Nebojsa S. Marinkovic, Eric A. Stach, Jason F. Weaver, Joanna Aizenberg, Philippe Sautet, Boris Kozinsky, Anatoly I. Frenkel

**Affiliations:** 1 Department of Materials Science and Chemical Engineering, Stony Brook University, Stony Brook, NY 11794 USA; 2 Department of Chemistry and Chemical Biology, Harvard University, Cambridge, MA 02138 USA; 3 Department of Chemical and Biomolecular Engineering, University of California, Los Angeles, Los Angeles, CA 90095 USA; 4 Harvard John A. Paulson School of Engineering and Applied Sciences, Harvard University, Cambridge, MA 02138 USA; 5 Department of Materials Science and Engineering, University of Pennsylvania, Philadelphia, PA 19104 USA; 6 Department of Physics, Harvard University, Cambridge, MA 02138 USA; 7 Department of Chemical Engineering, Columbia University, New York, NY 10027 USA; 8 Department of Chemical Engineering, University of Florida, Gainesville, FL 32611 USA; 9 Department of Chemistry and Biochemistry, University of California, Los Angeles, Los Angeles, CA 90095 USA; 10 Robert Bosch LLC, Research and Technology Center, Cambridge, MA 02139 USA; 11 Chemistry Division, Brookhaven National Laboratory, Upton, NY 11973 USA

**Keywords:** Catalytic mechanisms, Heterogeneous catalysis, Porous materials

## Abstract

Rational catalyst design is crucial toward achieving more energy-efficient and sustainable catalytic processes. Understanding and modeling catalytic reaction pathways and kinetics require atomic level knowledge of the active sites. These structures often change dynamically during reactions and are difficult to decipher. A prototypical example is the hydrogen-deuterium exchange reaction catalyzed by dilute Pd-in-Au alloy nanoparticles. From a combination of catalytic activity measurements, machine learning-enabled spectroscopic analysis, and first-principles based kinetic modeling, we demonstrate that the active species are surface Pd ensembles containing only a few (from 1 to 3) Pd atoms. These species simultaneously explain the observed X-ray spectra and equate the experimental and theoretical values of the apparent activation energy. Remarkably, we find that the catalytic activity can be tuned on demand by controlling the size of the Pd ensembles through catalyst pretreatment. Our data-driven multimodal approach enables decoding of reactive structures in complex and dynamic alloy catalysts.

## Abstract (web summary)

Rational catalyst design is crucial toward achieving more energy-efficient and sustainable catalytic processes. Here the authors report a data-driven approach for understanding catalytic reactions mechanisms in dilute bimetallic catalysts by combining X-ray absorption spectroscopy with activity studies and kinetic modeling.

## Introduction

In recent decades, worldwide energy demand has risen dramatically^1^, and nearly 30% of industrial energy use is tied to chemical production^2^ that relies heavily on heterogeneous catalysis. To increase the efficiency and sustainability of catalytic processes, multidisciplinary analytical strategies are required beyond the conventional trial-and-error approach to design more efficient catalysts^3^. Specifically, rational catalyst design aims to establish and apply fundamental structure-activity relationships at the nanoscale. Two central requirements are (i) the identification of the active sites and (ii) a theoretical framework for the simulation and prediction of reaction mechanisms and kinetics. Combined experimental characterizations and first-principles modeling have been employed to investigate predefined active site structures^4–6^. However, accurate identification of the active site remains a significant challenge due to the dynamic nature of surface composition and morphology evolving in time^7–9^.

The class of dilute alloy catalysts has tremendous industrial importance because they can enhance the activity and selectivity of chemical reactions while minimizing the use of precious metals^10–14^. Major advancements are sought in understanding the nature of their catalytically active sites^14^. In bimetallic nanoparticle systems, intra- and inter-particle heterogeneities give rise to a diverse population of sites and particle morphologies^15^. Furthermore, the active site can dynamically restructure under reaction conditions^16–19^. Although density functional theory (DFT) is widely used to investigate the thermodynamics of surface segregation in alloy systems^20–24^, the large computational cost precludes its use for direct sampling of the large underlying configurational space as well as the long timescale. As such, the relationship between the active site structure and the corresponding reaction pathways and kinetics remains hidden.

A useful tool for unlocking this relationship is an operando experiment, in which changes in structural and chemical features are measured simultaneously with the catalytic activity^25–27^. However, the small amount of active component in dilute alloys, compounded with low weight loading of the particles, require characterization tools with sufficient surface and chemical sensitivities. Among many scattering and absorption-based techniques, in situ X-ray absorption spectroscopy (XAS), in the form of extended X-ray absorption fine structure (EXAFS) and X-ray absorption near edge structure (XANES), has proven to be well-suited for studying dilute alloy catalysts^28–30^. In particular, the recently developed neural network-assisted XANES inversion method (NN-XANES) has enabled the extraction of local coordination numbers directly from XANES^31^. This technique makes the analysis of active sites in dilute alloys possible under reaction conditions, far exceeding the capabilities of conventional EXAFS fitting^28^. To the best of our knowledge, NN-XANES, while demonstrated to be useful for investigating the structure and local composition of bimetallic nanoparticles^28,32^, has not yet been applied to decoding active site structure and activity mechanisms in any functional nanomaterial systems.

**Fig. 1:**
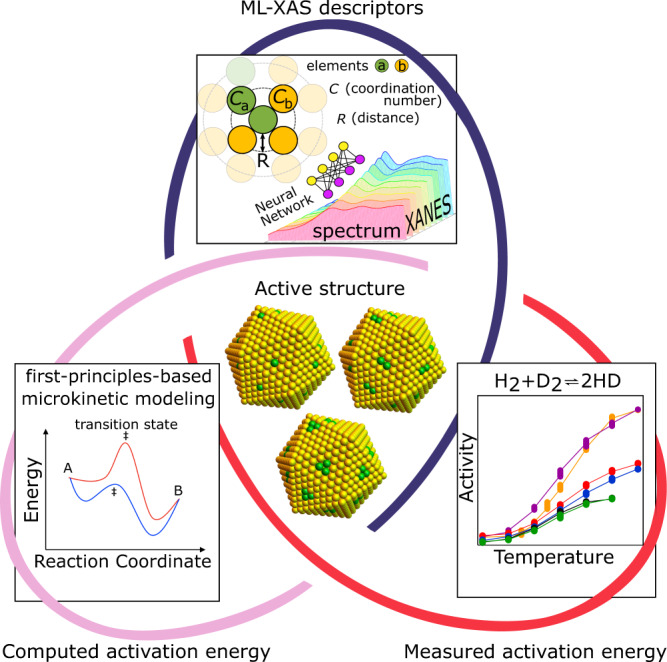
Multipronged strategy for decoding reactive structures. Measured X-ray absorption spectra are correlated directly to catalytic activity via theoretical modeling. (ML-XAS descriptors panel) Local structural descriptors, such as coordination number (C) and interatomic distance (R) between elements a (green) and b (yellow), are extracted from X-ray absorption spectra through machine learning (ML-XAS), via the Neural Network, to constrain the set of candidate active sites. (Computed activation energy panel) The corresponding reaction pathways, from states A to B through the transition state ‡, are modeled using first-principles-based microkinetic modeling. (Measured activation energy panel) Apparent activation energies from computations are compared to experimental measurements, where colors represent different reaction starting conditions of the hydrogen (H) deuterium (D) exchange reaction (H_2_ + D_2_ ⇋ 2HD), to further narrow down the dominant active sites. (Active structure panel) Three possible nanoparticle motifs are shown where the active sites are green and vary between 1 and 3 atoms.

Obtaining coordination numbers and other structural descriptors of a particular catalytic component is necessary but not sufficient to conclusively determine the active site geometry. To do so, two challenges must be resolved. First, one needs to decouple XAS signals originating from spectator species that contribute to the spectrum but not to the reaction. Second, given a set of possible active site geometries, more than one candidate structure may agree with the structural descriptors obtained from NN-XANES. We overcome these challenges by combining catalytic activity measurements, machine-learning enabled spectroscopic analysis, and first-principles-based kinetic modeling (Fig. 1). Through joint experimental and theoretical methods, we determine (i) the local structural descriptors of the catalyst and (ii) the apparent kinetic parameters of the reaction network. Both the structural and kinetic criteria must be satisfied to ascertain the dominant active site species. This multimodal approach is demonstrated for the prototypical HD exchange reaction on Pd_8_Au_92_/RCT-SiO_2_ (RCT = raspberry--colloid--templated), previously shown to have excellent catalytic performance and stability for this reaction^33^, CO oxidation^29^, and selective hydrogenation^30,34^. Remarkably, we find that the activity of HD exchange is determined by the size of surface Pd ensembles at the order of only a few atoms, which can be controlled directly through different catalyst treatments.

## Results and discussion

### Treatment alters the catalyst activity

The dilute alloy Pd_8_Au_92_/RCT-SiO_2_ catalyst was synthesized according to the established procedures^33^ (Methods). The catalyst is composed of 4.6 ± 0.6 nm (mean size) bimetallic nanoparticles with 8.3 at% Pd, embedded in RCT-SiO_2_. From electron microscopy and energy dispersive spectroscopy (EDS) measurements (Methods), the majority of Pd is homogeneously mixed with Au prior to any treatments and catalysis (Supplementary Fig. 1).

**Fig. 2:**
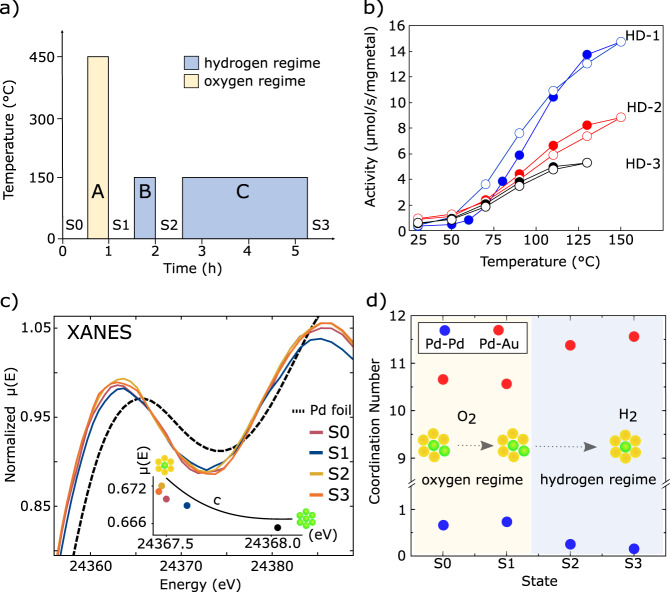
Pd_8_Au_92_/RCT-SiO_2_ is subjected to HD exchange reaction after three sequential pretreatments. **a** Initial state S0 undergoes O_2_ treatment to starting state S1 (treatment A), followed by H_2_ treatments to starting states S2 and then S3 (treatments B and C respectively). The hydrogen regime is shaded blue, and the oxygen regime is shaded yellow. **b** Activity versus temperature for three HD exchange experiments, labeled HD-1 to HD-3, corresponding to three different starting states (S1-S3). The solid and open circles indicate the heating and cooling regimes, respectively. **c** Normalized XANES collected for the different samples (pink = S0, blue = S1, yellow = S2, orange = S3, and dashed black = Pd foil). The inset shows a shift in the spectral center of mass “*c*”. **d** Coordination numbers (blue = Pd–Pd bonds and red = Pd–Au bonds) from NN-XANES are consistent with Pd atoms having more Pd neighbors after O_2_ treatment (yellow shade) and less Pd neighbors after H_2_ treatment (blue shade). Atomic Pd ensembles in (**c**) and (**d**) are depicted with bird’s-eye-view schematics (yellow = Au; green = Pd).

Previous experiments have established that treating the catalyst with O_2_ and H_2_ alters the activity^30^, implying treatment-induced changes of the catalyst surface structure. The exact kinetic mechanism of surface rearrangement in dilute alloys is still unknown, but it has long been established that oxidative environments result in a thermodynamic preference for Pd to reside on the surface of PdAu alloy nanoparticle and model surfaces^35,36^. To investigate the result of the treatment-induced restructuring of the surface, the HD exchange reaction was examined after three sequential treatments (A, B, C) of our sample (Fig. 2a; Methods). State 1 (S1) was obtained after O_2_ treatment at 500 °C for 30 min (treatment A), followed by State 2 (S2) after H_2_ treatment at 150 °C for 30 min (treatment B), followed by State 3 (S3) after another H_2_ treatment over 210 min with step-wise heating to 150 °C (treatment C). Throughout both heating and cooling phases, a steady-state HD formation rate was established at each temperature (Supplementary Fig. 3a–c).

The three starting states exhibited three distinct HD exchange reaction kinetics (numbered correspondingly) (Fig. 2b). The apparent activation energies (*E*_a_) and axis intercepts (*A*) were obtained from the Arrhenius analysis (Supplementary Fig. 3d, e). The HD-1 heating step exhibited the largest *E*_a_ = 0.67 ± 0.05 eV and *A* = 30. Starting with the HD-1 cooling phase, the values decreased rapidly, settling to *E*_a_ = 0.34 ± 0.05 eV and *A* = 18 by HD-2. The pronounced hysteresis between HD-1 heating and cooling suggests a change occured during the reaction in addition to that induced by the treatment. Overall, the changes in the kinetic parameters suggest that the number and the nature of the active species present in S1 have changed in S2 and S3. This hypothesis is further supported and elucidated by spectroscopy and theoretical modeling.

### Treatment restructures the catalyst

Pd *K*-edge XAFS spectra were collected (Methods) for the catalyst in the initial state (S0), as well as S1 to S3 after sequential treatments A through C, respectively (Fig. 2a). Treatment-induced changes in the XANES were observed, quantified by the shift in the spectral center of mass (Fig. 2c, inset). After treatment A with O_2_ (S0 to S1), the center of mass shifts toward the limit defined by the bulk Pd reference, and, after treatment B with H_2_ (S1 to S2)—away from the bulk Pd past S0, remaining at the same position after further treatment C with H_2_ (S2 to S3). These shifts are direct evidence of the structural changes induced by the catalyst treatments. The shift away from the bulk Pd upon H_2_ treatment has been attributed to an increase in the number of Pd-Au neighbors resulting from Pd dissolution into the Au host^28^.

To understand these structural changes more precisely, quantitative analysis was performed using partial coordination numbers (CNs) (${C}_{{{{{{\rm{Pd}}}}}}-{{{{{\rm{Pd}}}}}}}$ and ${C}_{{{{{{\rm{Pd}}}}}}-{{{{{\rm{Au}}}}}}}$) obtained from the NN-XANES inversion method^6^ (Fig. 2d; Methods) and conventional EXAFS fitting (Supplementary Fig. 4; Methods). Because of the relatively low sensitivity of EXAFS to the Pd–Pd contribution in dilute Pd limit, only a weak Pd-Pd contribution was detected in S0 and S1 but not in S2 and S3 (Supplementary Table 1). The NN-XANES analysis reveals the same trend in the CNs as the EXAFS fitting but with lower relative uncertainties, allowing us to detect Pd-Pd bonding at all regimes. In all samples, ${C}_{{{{{{\rm{Pd}}}}}}-{{{{{\rm{Pd}}}}}}}$ < 1.0, as expected for dilute alloys where Pd thermodynamically prefers to be fully coordinated with Au^30^.

From S0 to S1, treatment A with O_2_ slightly increases ${C}_{{{{{{\rm{Pd}}}}}}-{{{{{\rm{Pd}}}}}}}$ and decreases ${C}_{{{{{{\rm{Pd}}}}}}-{{{{{\rm{Au}}}}}}}$, consistent with mild segregation of Pd to the surface. In contrast, from S1 to S2, treatment B with H_2_ decreases ${C}_{{{{{{\rm{Pd}}}}}}-{{{{{\rm{Pd}}}}}}}$ from 0.73 to 0.25 and increases ${C}_{{{{{{\rm{Pd}}}}}}-{{{{{\rm{Au}}}}}}}$ from 10.57 to 11.38, consistent with Pd dissolution into the subsurface. Further evidence of dissolution is seen in the increase of the total Pd CN from 11.30 to 11.63. The full 12-fold coordination would correspond to 100% of Pd residing in the subsurface and being inaccessible for heterogeneous catalysis. The total Pd CN below 12 indicates the presence of some undercoordinated Pd on the surface. From S2 to S3, additional treatment C with H_2_ exhibits the same trend to a much lesser extent, i.e., ${C}_{{{{{{\rm{Pd}}}}}}-{{{{{\rm{Pd}}}}}}}$ decreases and the total Pd CN increases.

The extent of Pd mixing with Au was analyzed by comparing the ratio of coordination numbers ${C}_{{{{{{\rm{Pd}}}}}}-{{{{{\rm{Pd}}}}}}}:{C}_{{{{{{\rm{Pd}}}}}}-{{{{{\rm{Au}}}}}}}$ to the ratio of compositions ${x}_{{{{{{\rm{Pd}}}}}}}:{x}_{{{{{{\rm{Au}}}}}}}$ (0.083:0.917). In all states, ${C}_{{{{{{\rm{Pd}}}}}}-{{{{{\rm{Pd}}}}}}}:{C}_{{{{{{\rm{Pd}}}}}}-{{{{{\rm{Au}}}}}}}$ is less than ${x}_{{{{{{\rm{Pd}}}}}}}:{x}_{{{{{{\rm{Au}}}}}}}$, which corresponds to a tendency for Pd to disperse in Au, consistent with the EDS observations (Supplementary Fig. 1b, c). The dispersion tendency decreases after O_2_ treatment and increases after H_2_ treatment. These results are also consistent with DFT-computed segregation free energies (${G}_{{{{{{\rm{seg}}}}}}}$) of representative surface Pd ensembles in the presence of chemisorbed oxygen and hydrogen, referenced to gas-phase molecules and subsurface Pd monomers (Supplementary Fig. 5; Sec. 1, Supplementary Methods). Globally, Pd prefers to remain dispersed in the subsurface (${G}_{{{{{{\rm{seg}}}}}}}=0$), highlighting the metastable nature of these ensembles. The next favorable structure is the extended surface Pd oxide model^7^ (${G}_{{{{{{\rm{seg}}}}}}}=0.17\,{{{{{\rm{eV}}}}}}/{{{{{\rm{Pd}}}}}}$), considered as a limiting case of larger ensembles, but it is precluded from our reactivity modeling as it is inconsistent with the EELS map (Supplementary Fig. 1d) as well as the observed ${C}_{{{{{{\rm{Pd}}}}}}-{{{{{\rm{Pd}}}}}}} \, < \, 1.0$ across all samples. The precise mechanistic and kinetic relevance of such oxide phases remains beyond the scope of this work and would require much more advanced atomistic modeling approaches.

The relative stability of the ensembles is inverted upon chemisorption. Across all cases, O_2_ provides the largest thermodynamic driving force to form larger metastable ensembles (${G}_{{{{{{\rm{seg}}}}}}}=0 \sim 0.45\,{{{{{\rm{eV}}}}}}/{{{{{\rm{Pd}}}}}}$; Pd_5_O_4_ to trimer). Once O_2_ is removed after the initial pretreatment, these larger ensembles are expected to lower the surface free energy by fragmenting toward smaller-sized ensembles. Under H_2_, the same driving force for segregation becomes less pronounced (${G}_{{{{{{\rm{seg}}}}}}}=0.35 \sim 0.4\,{{{{{\rm{eV}}}}}}/{{{{{\rm{Pd}}}}}}$; Pd monolayer to trimer). Moreover, H_2_ chemisorption remains largely endergonic on Pd ensembles under the pretreatment condition (${G}_{{{{{{\rm{ads}}}}}}}=0 \sim 0.5\,{{{{{\rm{eV}}}}}}$; trimer to monomer), which would favor H_2_ desorption and partial dissolution of Pd into even smaller ensembles.

### Modeling resolves Pd ensemble reactivity

**Fig. 3:**
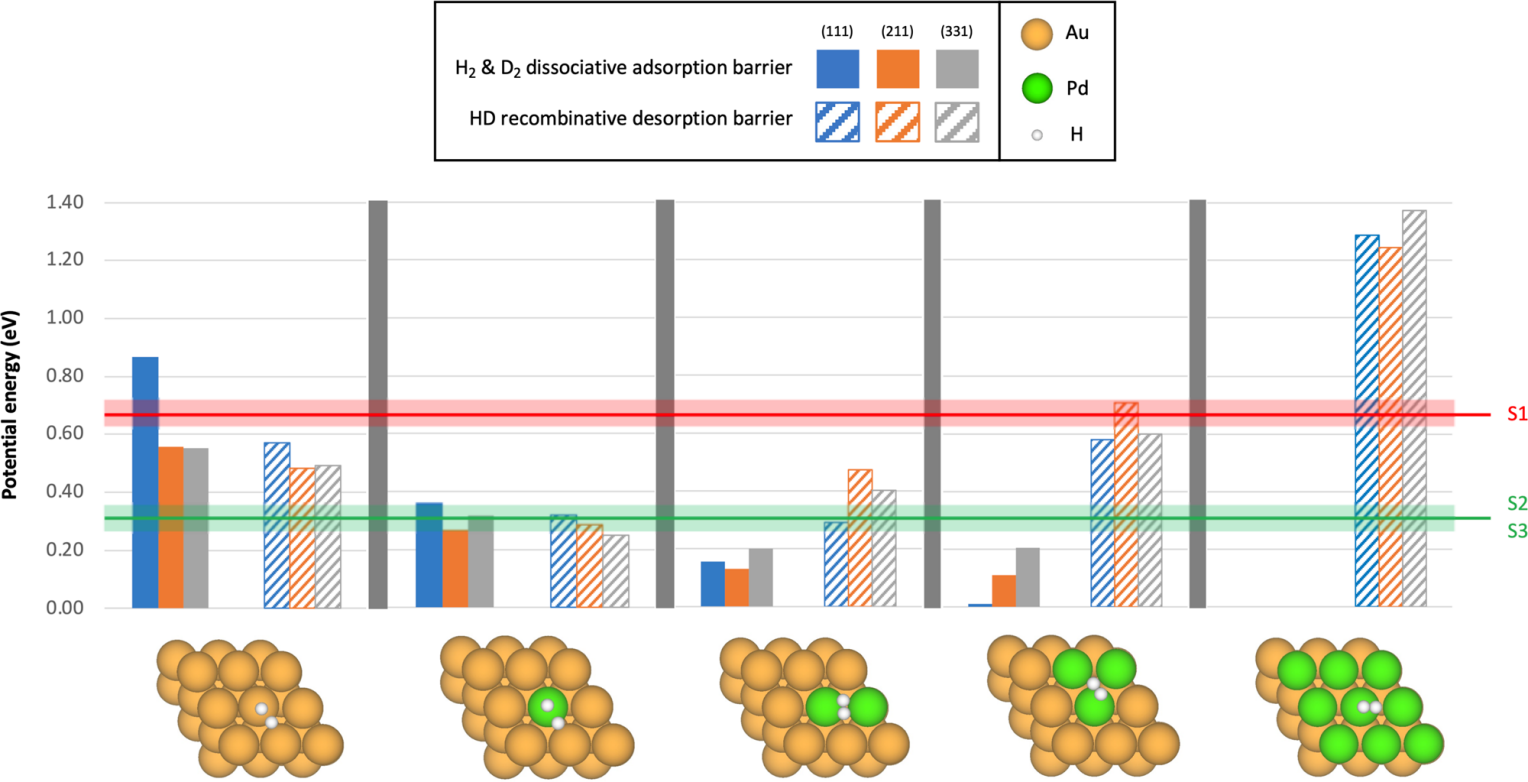
DFT modeling establishes the Sabatier optimum with dilute Pd ensembles. Three close-packed facets are considered: (111) for terrace; (211) and (331) for step edges. Five model surfaces are considered: pure Au, Pd monomer, dimer, trimer, and Pd monolayer. As indicated by the plotted energy barriers, there is a trade-off between H_2_/D_2_ dissociative adsorption (filled) and HD recombinative desorption (hatched) with increasing surface Pd content. Horizontal lines indicate experimentally measured apparent activation energies with uncertainties over shaded regions: the values for sample S1 (O_2_-treated) and S2/S3 (H_2_-treated) agree well with those computed for the Pd trimer and monomer/dimer, respectively. All DFT-computed values have an uncertainty of 0.05 eV.

To ascertain the atomic-level structure of the active sites, HD exchange reaction pathways were characterized via transition state modeling using DFT calculations (Supplementary Figs. 6–8; Methods). On the basis of the quantitative CN analysis that established dilute alloy motifs, several types of model surfaces with close-packed facets were considered. These structures enable systematic investigation of the effect of (i) the atomic arrangement of Pd in the active site (ensemble effect) and (ii) the local coordination environment of the active site (facet effect). The zero-point-corrected energy barriers associated with H_2_/D_2_ dissociative adsorption and HD recombinative desorption are shown in Fig. 3. Spillover and migration of atomic H/D are relatively facile and of secondary importance in determining the overall catalytic kinetics (Sec. 2, Supplementary Methods). Moreover, migration into the subsurface^37,38^ was not considered in our dilute systems, where Pd largely remains dispersed as isolated atoms in the interior of Au. A recent study of Pd/Ag(111) showed that subsurface H can become metastable only in the presence of locally extended Pd under high H_2_ pressures^39^.

The Sabatier optimum is clearly demonstrated by the dilute Pd ensembles, bounded by the chemisorption-limited Au surface on the left and the desorption-limited Pd monolayer on the right. As the surface Pd content increases, chemisorption becomes more facile, with barriers decreasing from ~0.3 to ~0.1 eV across Pd monomer to trimer, finally becoming barrierless on Pd monolayer which exhibits pure Pd-like behavior (Supplementary Fig. 8c). At the same time, desorption becomes more difficult, with barriers increasing from ~0.3 to ~0.6 eV across Pd monomer to trimer, finally exceeding 1.0 eV on Pd monolayer. This trade-off is driven by the ensemble effect, with minimal influence of the facet effect, except in the case of pure Au. Moreover, there is good agreement between the computed energy barriers and the experimentally measured apparent activation energies (Fig. 3; Supplementary Fig. 3d). Specifically, the measured apparent activation energies of 0.29–0.34 eV for S2 and S3 (H_2_-treated) agree well with the computed energy barriers of ~0.3-0.4 eV for Pd monomer and dimer, whereas the measured value of 0.67 eV for S1 (O_2_-treated) matches the computed value of ~0.65 eV for the Pd trimer. This trend is consistent with a previous study^33^ and proposes Pd trimers and monomers/dimers as compelling active site candidates upon O_2_- and H_2_-treatment, respectively.

**Fig. 4:**
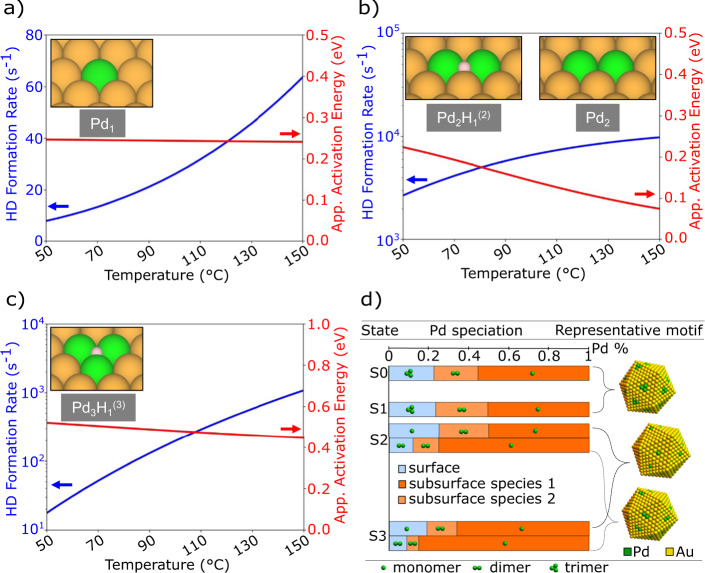
First-principles-based microkinetic modeling bridges theory and experiment. **a**–**c** Computed activity, the apparent activation energy (${E}_{{{{{{\rm{a}}}}}}}$), and most abundant intermediates (insets; superscript = H-Pd coordination number) of HD exchange reaction on dilute Pd ensembles. The activity increases in the order of Pd monomers, trimers, and dimers. At 50 °C, trimers have a higher ${E}_{{{{{{\rm{a}}}}}}}$ (0.52 eV) compared to monomers and dimers (0.20–0.25 eV). **d** Pd speciation (at.%) within the surface (blue) and subsurface/bulk (light and dark orange) responsible for the observed coordination numbers in the initial state (S0), and after pretreatments A (S1), B (S2), and C (S3). On the basis of theoretical modeling, surfaces of S0/S1 are constrained to trimers, and either monomers or dimers for S2 and S3 each. **a**–**d** Pd atoms are green and Au atoms are yellow.

To bridge theory and experiment, we performed microkinetic simulations using DFT-derived kinetic parameters as inputs (Methods; Sec. 3-4, Supplementary Methods). This method circumvents the previously employed assumption of a single rate-limiting step^33^. Pd dimers have the highest activity for HD exchange, with a low apparent barrier of 0.22 eV at 50  °C (Fig. 4b). Although Pd monomers have a similarly low apparent barrier of 0.25 eV, their activity is lower by ~2 orders of magnitude due to the greater required loss of entropy of gas-phase H_2_/D_2_^33^ (Fig. 4a). Due to more difficult desorption, Pd trimers exhibit an intermediate level of activity, with a higher apparent activation energy of 0.52 eV at 50 °C (Fig. 4c). Although the degree of rate control analysis shows that there is no single rate-limiting transition state in all three cases, the transition states of D_2_ dissociation and HD recombination control the rate of H/D exchange over Pd monomers/dimers (Supplementary Fig. 9a–f), and desorption of the HD molecule controls the rate of H/D exchange over Pd trimers (Supplementary Fig. 9g–i), consistent with the DFT energetics of Fig. 3. These observations reinforce the catalytic predominance of Pd trimers in O_2_-treated samples, in contrast to Pd monomers/dimers in H_2_-treated samples.

### Treatment controls the Pd distribution

To quantitatively analyze the distribution of Pd ensembles in terms of the structural descriptors obtained from NN-XANES, we parametrized the partial CNs in terms of the distribution of Pd in Au, i.e., the number of Pd monomers, dimers, and trimers on a model catalyst surface and in the interior (Methods). Figure 4d summarizes the distribution of the Pd ensembles responsible for the observed CNs in samples S0-S3 using a representative icosahedral Au nanoparticle model of size 4.6 nm (in agreement with the TEM measurements). The distributions were obtained with constraints on the type of surface species present, as inferred from theoretical modeling. Samples S0 and S1 (calcined in air and O_2_-treated, respectively) are characterized by a surface consisting of Pd trimers and an interior of monomers and dimers. In contrast, the H_2_-treated samples S2 and S3 are characterized by a surface consisting of a small number of Pd dimers and/or monomers with an interior dominated by monomers. The surface Pd content decreases from S2 to S3, consistent with H_2_-induced dissolution of Pd. This numerical analysis confirms that the dilute Pd ensembles satisfy both the kinetic and structural criteria of the active site.

A multipronged strategy has been developed to resolve the active site structure of a dilute bimetallic catalyst at the atomic level, a central requirement for advancing rational catalyst design. By combining catalysis, machine learning-enabled spectroscopic analysis, and first-principles-based kinetic modeling, we demonstrate the effect of catalyst treatment on its nanostructure, active site distribution, and reactivity toward the prototypical HD exchange reaction. A dilute Pd-in-Au alloy supported on raspberry-colloid-templated SiO_2_ was chosen for its excellent catalytic performance and stability^29,30,33,34^. Upon H_2_ treatment, the activity and the apparent activation energy decreased significantly. These observations are attributed to treatment-induced catalyst restructuring, quantitatively analyzed in terms of the coordination numbers extracted from neural network-assisted inversion of the X-ray absorption spectra^28^. The majority of Pd remained dispersed inside the Au host, with a small amount of Pd segregating toward the surface upon O_2_ treatment and dissolving into the bulk upon H_2_ treatment.

On the basis of this motif, theoretical modeling of the reaction network on several model surfaces has established dilute Pd ensembles as the catalytically predominant active sites. These ensembles numerically correspond to the observed coordination numbers, thereby satisfying both the structural and kinetic criteria of the active site. Remarkably, the reactivity is tuned by modulating the active site on the order of only a few atoms (*n* = 1–3) through catalyst treatment. Our multidisciplinary approach considerably narrows down the large configurational space to enable precise identification of the active site and can be applied to more complex reactions in related dilute alloy systems, such as selective hydrogenation of alkynes^34,40^ and CO oxidation^29^.

## Methods

### Catalyst synthesis

For the RCT (raspberry-colloid-templated) synthesis, we refer to the procedure reported by van der Hoeven et al.^33^. The gold nanoparticles were prepared using a procedure described by Piella et al.^41^ on a 450 mL scale at 343 K. The reaction mixture contained 0.3 mL 2.5 mM tannic acid, 3.0 mL 150 mM K_2_CO_3_, and 3.0 mL 25 mM HAuCl_4_ in H_2_O. The raspberry colloids were prepared by attaching the gold nanoparticles to the sacrificial polystyrene (PS) colloids (d_PS_  = 393 nm). To 150 mL AuNPs, 1.5 mL aqueous PVP solution (0.1 g PVP per mL H_2_O) and 12 mL thiol-functionalized PS colloids (5.0 wt% in water) were added. After washing three times with MQ H_2_O the colloids were redispersed in 12 mL MilliQ H_2_O. The Pd growth on the AuNPs attached to polystyrene colloids was done at low pH to ensure sufficiently slow reaction rates and selective growth on the AuNPs^42^. To 12 mL raspberry colloid dispersion (5.0 wt% PS in water), 150 mL in MQ H_2_O, 1.5 mL 0.1 M HCl, 270 µL 10 mM Na_2_PdCl_4_ and 270 µL 40 mM ascorbic acid were added to obtain the Pd_8_Au_92_ NPs. The raspberry colloids were washed twice, redispersed in 12 mL MQ H_2_O, and dried at 65 °C in air. Next, the colloidal crystal was infiltrated with a pre-hydrolyzed TEOS solution (33 vol% of a 0.10 M HCl in H_2_O solution, 33 vol% ethanol, 33 vol% TEOS). Finally, the samples were calcined to remove PS colloids by heating them in static air from room temperature to 773 K with 1.9 K/min and held at 773 K for 2 h. Inductively coupled plasma mass spectrometry (ICP-MS, Agilent Technologies 7700x) was used for compositional analysis (metal composition and metal weight loading). The exact composition is 8.3 at% Pd, 4.4 wt% total metal loading.

### HD exchange experiments

Catalysis experiments were performed using the same flow cell as the Synchrotron in situ XAS experiments to directly correlate the structural changes observed in XAS with the activity of the sample toward HD exchange. A total of three HD exchange experiments were performed for the Pd_8_Au_92_ sample after three different sequential treatments (A, B, C) (Fig. 2a). Treatment A consisted of 30 min heating at 500 °C in 20% O_2_ atmosphere (balance Ar); treatment B consisted of 30 min heating at 150 °C in 25% H_2_ atmosphere (balance Ar); and treatment C consisted of the same H_2_ treatment for 210 min, with temperatures maintained at 100, 120, 140, 120, 100 °C in sequence for 30 min at each temperature to allow full equilibration of the structural changes induced by the treatment. The temperature was increased and decreased at a rate of 10 °C/min.

The three sequential treatments resulted in three distinct starting states: State 1 (S1), State 2 (S2), and State 3 (S3) after treatment A, B, and C, respectively. In HD reaction 1 (HD-1) with starting state S1, HD exchange was monitored during heating (HD-1 heating) and cooling (HD-1 cooling) stages (Fig. 2b). The same procedure applies for HD reactions 2 and 3 (HD-2 and HD-3, respectively).

Each treatment resulted in different HD exchange activities, so the sample amount was varied to keep the conversion well below 50% (maximum conversion in the reaction with a statistical mixture of 1H_2_:1D_2_:1HD) in the entire temperature range so as to accurately measure the catalytic performance (Supplementary Fig. 3e). The undiluted sample was loaded into quartz capillary of internal diameter 1 mm. The ends of the sample bead were blocked with quartz wool to avoid the powder displacement in the gas flow. The reactions were performed in the gas mixture of 12.5% H_2_, 12.5% D_2_, 72% Ar, and 3% N_2_, with a total flow of 15 mL/min. The temperature was increased between 30 °C and 150 °C with 10/20 °C steps and kept at each step for 5–30 min to achieve equilibrium (Supplementary Fig. 3a–c). The reaction products were measured with an online mass spectrometer (RGA, Hiden Analytical).

The MS signals of H_2_ and D_2_ changed upon consumption, forming the basis of extracting the HD formation rate (Fig. 2b) and the apparent activation energy from the Arrhenius analysis (Supplementary Figs. 2, 3). The baseline signal, indicating the sensitivity of the MS toward H_2_/D_2_/HD, was obtained from the bypass. The conversion was calculated using both H_2_ and D_2_ signals from which the average activity was extracted. The activity values in the range of 1–20% were used from each temperature step for the Arrhenius analysis.

### Transmission electron microscopy (TEM) and energy-dispersive X-ray spectroscopy (EDS)

TEM was performed with a JEOL NEOARM operating at 200 kV. All images are high-angle annular dark-field scanning transmission electron microscopy (HAADF-STEM) data. The diameter of the used condenser lens aperture was 40 μm, the probe current was 150 pA, and the camera length was 4 cm. EDS was performed with two detectors provided by JEOL Ltd. and the maps were obtained with DigitalMicrograph, a software developed by Gatan Inc.

### In situ X-ray absorption fine structure spectroscopy (XAS)

XAS experiments at the Pd *K*-edge were carried out at the QAS beamline of the NSLSII. Ten milligrams of sample was loaded into a borosilicate capillary. The sample treatments were identical to those performed for activity measurements: treatment A was performed with O_2_ (500 °C, 20% O_2_ balance He, 30 min, cooled down in O_2_ to room temperature for data collection); treatment B with H_2_ (150 °C, 25% H_2_ balance N_2_, 30 min, cooled down in H_2_ to room temperature for data collection); and treatment C with H_2_ and slow step-wise heating up to 140 °C (25% H_2_ balance N_2_, 30 min, cooled down in H_2_ to room temperature for data collection). The entire experiment, including treatments and data collection steps, was performed with 15 scc gas flow. Data were collected at room temperature after each treatment. Each step was equilibrated for at least 30 min.

### Extended X-ray absorption fine structure (EXAFS) analysis

The analysis of EXAFS data was performed with IFEFIT package (Supplementary Fig. 4)^43^. The S_0_^2^ amplitude reduction factor value was obtained from the fitting of Pd foil that was previously measured at the same beamline. The obtained value of 0.78 was used in all subsequent fittings of S0-S3 spectra. For the fitting of S0 & S1 data, two nearest-neighbor photoelectron paths Au–Au and Pd–Au were chosen. For S2 & S3, only Au–Au path was chosen for the fitting, as adding Pd–Au path made the fit unstable. Fitting *k* range values were: 2–12 Å^−1^, 2–11 Å^−1^, 2–9 Å^−1^, and 2–10 Å^−1^ for S0-S3, respectively. Fitting *R* range values were 1.75–3.7 Å, 1.4–3.5 Å, 1.45–3.5 Å, and 1.4–3.6 Å for S0-S3, respectively. Both *k* and *R* fitting ranges were optimized for each dataset separately, in order to minimize the standard deviations. The best-fitting results are summarized in Supplementary Table 1.

### Neural network-assisted X-ray near edge structure inversion analysis (NN-XANES)

The development and validation of the NN-XANES inversion method for PdAu bimetallic nanoparticles are reported in our previous work^28^. Before the application of the trained NN object in Mathematica 12, the experimental data were preprocessed with Athena. The catalyst Pd *K*-edge spectra of S0-S3 were aligned, edge step normalized, and then interpolated to a 95 point non-uniform energy mesh that spanned energies from *E*_min_ = 24339.8 eV to *E*_max_ = 24416.6 eV with a step size of 0.6 eV for data points near the absorption edge, which gradually increased to 1.7 eV for points approaching *E*_max_. The same procedure was completed for a Pd *K*-edge spectrum of Pd foil that was collected at the same time as the catalyst spectra. The normalized and interpolated Pd foil spectrum was subtracted from the normalized and interpolated spectra of S0-S3. Finally, the energies and absorption coefficients were normalized between 0 and 1 using the commonly used min-max normalization procedure:

1 $$Z=\frac{X-{{\min }}(X)}{{{\max }}(X)-{{\min }}(X)}.$$

Here *X* is the training data input, min(*X*) is the smallest input, max(*X*) is the largest input, and *Z* is the normalized input. To estimate relative errors in the coordination number predictions, 10 independently trained NNs were applied to the processed data^28,31^. The coordination numbers and their uncertainties are presented in Supplementary Table 1.

### Density functional theory (DFT)

We perform DFT calculations using plane-wave basis sets and the projector augmented-wave (PAW) method^44^ as implemented in the Vienna Ab Initio Simulation Package (VASP)^45^. The plane-wave kinetic energy cutoff is set at 450 eV. The Methfessel-Paxton smearing scheme^46^ is employed with a broadening value of 0.2 eV. All structures are optimized via ionic relaxation, with the total energy and forces converged to 10^−5 ^eV and 0.02 eV/Å, respectively. Gas-phase species are optimized in a 14 × 15 × 16 Å^3^ cell at the Γ-point with spin polarization. Lattice constants of bulk face-centered cubic Au and Pd are optimized according to the third-order Birch-Murnaghan equation of state, using a 19 × 19 × 19 **k**-point grid. We maintain the lattice constant of pure Au for all Pd-doped Au systems, given the dilute concentration of Pd in our model systems. All slab models are spaced by 16 Å of vacuum along the direction normal to the surface in order to avoid spurious interactions between adjacent unit cells. We fix the bottom layer(s) at bulk positions to mimic bulk properties. Supplementary Table 2 describes the computational set-up for the slab models of close-packed surfaces considered in our study.

We employ the Perdew-Burke-Ernzerhof (PBE) parametrization^47^ of the generalized gradient approximation (GGA) of the exchange-correlation functional. PBE provides Au and Pd lattice constants of 4.16 and 3.94 Å, within <0.1 Å of the experimental benchmark of 4.08 and 3.88 Å, respectively^48^. We also examine the dissociative adsorption energy of H_2_ on Pd(111), defined as the change in energy from an isolated slab and a gas-phase H_2_ to a combined system interacting as atomic H adsorbed on the slab:

2 $${E}_{{{{{{\rm{ads}}}}}}}=E\left[{{{{{{\rm{H}}}}}}}_{({{{{{\rm{ads}}}}}})}/{{{{{\rm{Pd}}}}}}(111)\right]-E\left[{{{{{\rm{Pd}}}}}}\left(111\right)\right]-\frac{1}{2}E\left[{{{{{{\rm{H}}}}}}}_{2\left({{{{{\rm{g}}}}}}\right)}\right].$$

PBE provides H adsorption energy of −0.57 eV at the low-coverage limit, within 0.1 eV of the experimental benchmark of −0.47 eV^49^. Based on these observations, we conclude that PBE is an appropriate standard reference functional that can provide a reasonable comparison with experiments for H_2_ chemisorption on Pd/Au systems.

We perform transition state modeling using the VASP Transition State Tools (VTST). Transition state pathways are first optimized via the climbing-image nudged elastic band (CI-NEB) method^50^, using three intermediate images generated by linear interpolation with a spring constant of 5 eV/Å^2^. The total forces, defined as the sum of the spring force along the chain and the true force orthogonal to the chain, are converged to 0.05 eV/Å. Then, the image with the highest energy is fully optimized to a first-order saddle point via the dimer method^51^, this time converging the total energy and forces to 10^−7 ^eV and 0.01 eV/Å, respectively. We confirm that the normal modes of all transition states contain only one imaginary frequency by calculating the Hessian matrix within the harmonic approximation, using central differences of 0.01 Å at the same level of accuracy as the dimer method.

Vibrational frequencies associated with geometrically inequivalent configurations of all isotopic species (H_2_, D_2_, HD, H, D) are obtained by calculating the Hessian matrix in a similar manner. Given the large difference in the masses of the adsorbates and the metal substrate, only the adsorbate degrees of freedom are considered in the calculations.

Zero-point energy corrections (ZPC) are applied to all dissociative adsorption and recombinative desorption processes by correcting the electronic energies of the corresponding initial, transition, and final states with their respective zero-point energies:

3 $${E}_{{{{{{\rm{ZPC}}}}}}}=\frac{1}{2}\mathop{\sum}\limits_{i}h{\nu }_{i}.$$

Here, $h$ is the Planck constant and ${\nu }_{i}$ is the non-imaginary vibrational frequency of normal mode $i$.

Conversion from the electronic energy at 0 K (${E}_{{{{{{\rm{DFT}}}}}}}$) to the ideal-gas Gibbs free energy ($G$) at a given temperature $T$ and pressure $P$ is given by:

4 $$G\left(T,P\right)={E}_{{{{{{\rm{DFT}}}}}}}+{E}_{{{{{{\rm{ZPC}}}}}}}+{k}_{{{{{{\rm{B}}}}}}}T+{\int }_{0}^{T}{c}_{V}{dT}-T\cdot S\left(T,P\right)$$

where ${c}_{V}$ is the constant-volume heat capacity. See Supplementary Table 3 for the statistical thermodynamics expressions of the integrated heat capacity and the entropy. Translational and rotational degrees of freedom are included for gas-phase molecules only.

### Microkinetic modeling

The microkinetic models were parameterized using the energetics of the H/D exchange reaction network, computed with DFT using the PBE functional (Methods). For a dilute Pd/Au alloy, the effect of H coverage on reaction energetics can be considered to be small because the Pd sites are far apart. On Pd(111), the H coverage also does not significantly impact the adsorption energy^52^. The rate constant of an elementary step is given by the Eyring equation:

5 $$k=\frac{{k}_{{{{{{\rm{B}}}}}}}T}{h}{{\exp }}\left(-\frac{{\triangle G}^{o{{\ddagger}} }}{{k}_{{{{{{\rm{B}}}}}}}T}\right)$$

where ${k}_{{{{{{\rm{B}}}}}}}$ is the Boltzmann constant, $h$ is the Planck constant, $T$ is the temperature, and ${\triangle G}^{o{{\ddagger}} }$ is the Gibbs free energy of activation at standard pressure.

The rate constant of adsorption for species $i$ is given by the collision theory:

6 $${k}_{{{{{{\rm{ads}}}}}},i}=\frac{\sigma {AP}^\circ }{\sqrt{2\pi {m}_{i}{k}_{{{{{{\rm{B}}}}}}}T}}{{\exp }}\left(-\frac{\triangle {E}_{{{{{{\rm{ads}}}}}}}^{{{\ddagger}} }}{{k}_{{{{{{\rm{B}}}}}}}T}\right)$$

where $\sigma$ is the sticking coefficient, $A$ is the surface area of the Pd ensemble, $P^\circ$ is the standard pressure (1 bar), ${m}_{i}$ is the mass of the adsorbate, and $\triangle {E}_{{{{{{\rm{ads}}}}}}}^{{{\ddagger}} }$ is the activation energy of adsorption. In this work, the sticking coefficient is set to 1, and the molecular adsorption process is taken to be barrierless. The surface areas are calculated using the bulk lattice constants of Pd and Au optimized with the PBE functional (3.94 and 4.16 Å, respectively). The atomic fraction of Pd in the alloy is set to 10%, in line with the concentration of 8% in the experimental samples. From Vegard’s law, the area occupied by one atom on (111) facet is $7.41\times {10}^{-20}\,{{{{{{\rm{m}}}}}}}^{2}$, which is then multiplied by the number of Pd atoms in each ensemble.

The corresponding rate constant for desorption is given by

7 $${k}_{{{{{{\rm{des}}}}}},i}=\frac{{k}_{{{{{{\rm{ads}}}}}},i}}{{K}_{{{{{{\rm{ads}}}}}},i}}$$

with the equilibrium constant of adsorption, ${K}_{{{{{{\rm{ads}}}}}},i}$:

8 $${K}_{{{{{{\rm{ads}}}}}},i}={{\exp }}\left(-\frac{{\triangle G}_{{{{{{\rm{ads}}}}}},i}^{^\circ }}{{k}_{{{{{{\rm{B}}}}}}}T}\right)$$

where ${\triangle G}_{{{{{{\rm{ads}}}}}},i}^{^\circ }$ is the Gibbs free energy of adsorption at standard pressure. The translational, rotational, and vibrational degrees of freedom fare considered for gaseous species, whereas only the vibrational degrees of freedom are included for surface intermediates and transition states. All vibrational frequencies below 100 cm^−1^ are rounded up to 100 cm^−1^.

The rate of elementary step $j$ was computed as follows:

9 $${r}_{j}={k}_{j}^{{{{{{\rm{fwd}}}}}}}\mathop{\prod}\limits_{i}{\alpha }_{i,{{{{{\rm{IS}}}}}}}^{{\nu }_{{ij}}^{{{{{{\rm{fwd}}}}}}}}\mathop{\prod}\limits_{i}{\alpha }_{i,{{{{{\rm{gas}}}}}}}^{{\nu }_{{ij}}^{{{{{{\rm{fwd}}}}}}}}-{k}_{j}^{{{{{{\rm{rev}}}}}}}\mathop{\prod}\limits_{i}{\alpha }_{i,{{{{{\rm{IS}}}}}}}^{{\nu }_{{ij}}^{{{{{{\rm{rev}}}}}}}}\mathop{\prod}\limits_{i}{\alpha }_{i,{{{{{\rm{gas}}}}}}}^{{\nu }_{{ij}}^{{{{{{\rm{rev}}}}}}}}.$$

Here, ${k}_{j}^{{{{{{\rm{fwd}}}}}}}$ and ${k}_{j}^{{{{{{\rm{rev}}}}}}}$ are the forward and reverse rate constants, and ${\nu }_{{ij}}^{{{{{{\rm{fwd}}}}}}}$ and ${\nu }_{{ij}}^{{{{{{\rm{rev}}}}}}}$ are the stoichiometric coefficients of reactant $i$ in the forward and reverse directions, respectively. The activity ${\alpha }_{i}$ is taken as the surface coverage fraction ${\theta }_{i}$ for intermediate states (labeled IS; including bare sites) and as the ratio of the partial pressure to the standard pressure, ${P}_{i}/P^\circ$, for gaseous species^53^.

The time-dependent coverages of surface intermediates are obtained as the steady-state solution of the following system of ordinary differential equations:

10 $$\frac{d{\theta }_{i}}{{dt}}=-\mathop{\sum}\limits_{j}{\nu }_{{ij}}^{{{{{{\rm{fwd}}}}}}}{r}_{j}+\mathop{\sum}\limits_{j}{\nu }_{{ij}}^{{{{{{\rm{rev}}}}}}}{r}_{j}$$

Following Wang et al.^54^, the steady-state solution is achieved in two steps. Starting from a bare surface, the equations are first integrated over 50 s until they have approximately reached a steady state. The resulting coverages are then used as an initial guess for numerical solution as follows:

11 $$0=-\mathop{\sum}\limits_{j}{\nu }_{{ij}}^{{{{{{\rm{fwd}}}}}}}{r}_{j}+\mathop{\sum}\limits_{j}{\nu }_{{ij}}^{{{{{{\rm{rev}}}}}}}{r}_{j}$$

12 $${\theta }_{{{{{{{\rm{Pd}}}}}}}_{n}}\left(t=0\right)=\mathop{\sum}\limits_{i}{\theta }_{{{{{{{\rm{Pd}}}}}}}_{n},i}$$

13 $$1=n\mathop{\sum}\limits_{i}{\theta }_{{{{{{{\rm{Pd}}}}}}}_{n},i}+\mathop{\sum}\limits_{i}{\theta }_{{{{{{\rm{Au}}}}}},i}$$

Here, ${\theta }_{{{{{{{\rm{Pd}}}}}}}_{n},{i}}$ and ${\theta }_{{{{{{\rm{Au}}}}}},{i}}$ are the surface coverages of species *i* on Pd_*n*_ and Au sites, respectively, and *n* is the number of Pd atoms in the ensemble.

The steady-state rates of HD formation over Pd_*n*_/Au(111) are solved at temperatures of 25–150 °C. The partial pressures of H_2_ and D_2_ are set to 9.9 kPa and that of HD to 0.2 kPa, with a balance of inert for the total pressure of 100  kPa. The reaction pathways are analyzed by computing the apparent activation energy, steady-state intermediate coverages, and the degrees of rate control for all surface intermediates and transition states^55^. The derivatives are evaluated numerically using step sizes of 0.1 °C and ${10}^{-4}\,{{{{{\rm{eV}}}}}}$ for the apparent activation energy and the degree of rate control, respectively.

### Coordination number parameterization

For a bimetallic system of Pd and Au atoms, the average first nearest neighbor coordination numbers from the Pd perspective can be represented as the vector $\{{\widetilde{C}}_{{{{{{\rm{Pd}}}}}}},{\widetilde{C}}_{{{{{{\rm{Au}}}}}}}\}$, where ${\widetilde{C}}_{{{{{{\rm{Pd}}}}}}}$ is the average Pd–Pd coordination number and ${\widetilde{C}}_{{{{{{\rm{Au}}}}}}}$ is the average Pd–Au coordination number. In general, the first nearest neighbor coordination numbers can be parametrized in terms of the Pd speciation with the following system of equations:

14 $$\left[\begin{array}{c}{\widetilde{C}}_{{{{{{\rm{Pd}}}}}}}\\ {\widetilde{C}}_{{{{{{\rm{Au}}}}}}}\end{array}\right]=\frac{1}{N}\left[\begin{array}{ccc}{\tilde{C}}_{{{{{{{\rm{Pd}}}}}}}_{1}} & \cdots & {\tilde{C}}_{{{{{{{\rm{Pd}}}}}}}_{i}}\\ {\tilde{C}}_{{{{{{{\rm{Au}}}}}}}_{1}} & \cdots & {\tilde{C}}_{{{{{{{\rm{Au}}}}}}}_{i}}\end{array}\right]\left[\begin{array}{ccc}{N}_{{{{{{\rm{Pd}}}}}}}^{(1)} & \cdots & 0\\ \vdots & \ddots & \vdots \\ 0 & \cdots & {N}_{{{{{{\rm{Pd}}}}}}}^{(i)}\end{array}\right]\left[\begin{array}{c}{s}_{1}\\ \vdots \\ {s}_{i}\end{array}\right]$$

where ${s}_{i}$ refers to the speciation, i.e. the number of occurrences of a specific structural configuration of Pd (e.g. surface monomer, dimer, etc.), ${N}_{{{{{{\rm{Pd}}}}}}}^{(i)}$ is the number of Pd atoms that are part of the motif ${s}_{i}$, ${\tilde{C}}_{{{{{{{\rm{Pd}}}}}}}_{i}}$ and ${\tilde{C}}_{{{{{{{\rm{Au}}}}}}}_{i}}$ are the partial Pd–Pd and Pd–Au coordination numbers of each Pd atom in ${s}_{i}$, respectively, and *N* is the total number of Pd atoms in the nanoparticle, i.e., $N={x}_{{{{{{\rm{Pd}}}}}}}\cdot {N}_{{{{{{\rm{tot}}}}}}}$, where ${x}_{{{{{{\rm{Pd}}}}}}}$ is the atomic ratio of Pd and ${N}_{{{{{{\rm{tot}}}}}}}$ is the total number of atoms in the particle.

To investigate the effect of surface monomers, dimers, and trimers, as well as subsurface dimers and monomers, we modify the system of equations:

15 $$\left[\begin{array}{c}{\widetilde{C}}_{{{{{{\rm{Pd}}}}}}}\\ {\widetilde{C}}_{{{{{{\rm{Au}}}}}}}\end{array}\right]=\frac{1}{N}\,\left[\begin{array}{ccccc}0 & 1 & 2 & 1 & 0 \\ 9 & 8 & 7 & 11 & 12\end{array}\right]\,\left[\begin{array}{ccccc}1 & 0 & 0 & 0 & 0\\ 0 & 2 & 0 & 0 & 0\\ 0 & 0 & 3 & 0 & 0\\ 0 & 0 & 0 & 2 & 0\\ 0 & 0 & 0 & 0 & 1\end{array}\right]\,\left[\begin{array}{c}{N}_{{{{{{\rm{m}}}}}}}\\ {N}_{{{{{{\rm{d}}}}}}}\\ {N}_{{{{{{\rm{t}}}}}}}\\ {N}_{{{{{{\rm{sd}}}}}}}\\ {N}_{{{{{{\rm{b}}}}}}}\end{array}\right]\,$$

where ${N}_{{{{{{\rm{m}}}}}}}$, ${N}_{{{{{{\rm{d}}}}}}}$, ${N}_{{{{{{\rm{t}}}}}}}$, ${N}_{{{{{{\rm{sd}}}}}}}$, and ${N}_{{{{{{\rm{b}}}}}}}$ are the numbers of surface monomers, surface dimers, surface trimers, subsurface dimers, and subsurface (bulk) monomers. The number of Pd atoms in each species are 1, 2, 3, 2, and 1, respectively, and the partial coordination numbers are determined by assuming a (111) surface orientation with bulk face-centered cubic packing. The assumption of (111) facet is backed by DFT calculations showing that exposed Pd atoms are thermodynamically more stable at close-packed terrace sites. The total number of Pd atoms is estimated as $N={x}_{{{{{{\rm{Pd}}}}}}}\cdot {N}_{{{{{{\rm{tot}}}}}}}$, where ${x}_{{{{{{\rm{Pd}}}}}}}$ is obtained from ICP-MS (0.083) and ${N}_{{{{{{\rm{tot}}}}}}}$ from TEM (4.6 $\pm$ 0.8 nm) as follows:

16 $${N}_{{{{{{\rm{tot}}}}}}}=\frac{1}{3}\left(2n+1\right)(5{n}^{2}+5n+3)$$

Here, $n$ is the side length of an icosahedron, and *n* = 8.4 for an icosahedron that is 4.6 nm, resulting in 2360 total atoms. We use the equation for an icosahedron because the original Au nanoparticles are icosahedra, and they are doped with a dilute amount of Pd which we assume does not substantially distort the morphology of the nanoparticle.

## Supplementary information

Supplementary Information

## Data Availability

All data generated or analyzed during this study are included in this published article (and its supplementary information files).
