# Supplementary material for: Decoding reactive structures in dilute alloy catalysts
Source: Nat Commun. 2022 Feb 11;13:832. doi: 10.1038/s41467-022-28366-w (PMC8837610; doi:10.1038/s41467-022-28366-w)
Supplement: Supplementary file 1 — Supplementary Information [file 41467_2022_28366_MOESM1_ESM.pdf]

Supplementary Information for

Decoding Reactive Structures in Dilute Alloy Catalysts

Nicholas Marcella[1*], Jin Soo Lim[2*], Anna M. Płonka[1*], George Yan[3*], Cameron J. Owen[2], Jessi E. S. van der Hoeven[2,4], Alexandre C. Foucher[5], Hio Tong Ngan[3], Steven B. Torrisi[6], Nebojsa S. Marinkovic[7], Eric A. Stach[5], Jason F. Weaver[8], Joanna Aizenberg[2,4], Philippe Sautet[3,9], Boris Kozinsky[4,10]✉, and Anatoly I. Frenkel[1,11]✉

Correspondence to: bkoz@seas.harvard.edu; anatoly.frenkel@stonybrook.edu

This PDF file includes:

Supplementary Methods

Supplementary Tables

Supplementary Figures

Supplementary References

Supplementary Methods

1. DFT calculation: Surface segregation of Pd/Au(111) with O_2 and H_2 chemisorption

We investigate the relative stability of surface Pd ensembles with and without chemisorbed oxygen and hydrogen. We first examine the dissociative adsorption energy of O_2 on Pd(111), defined as:

$$E_{\mathrm{ads}} = E\left[\mathrm{O}_{(\mathrm{ads})}/\mathrm{Pd}(111)\right] - E[\mathrm{Pd}(111)] - \frac{1}{2}E\left[\mathrm{O}_{2(\mathrm{g})}\right] \quad (1)$$

PBE provides O adsorption energy of –1.41 eV at the low-coverage limit, overbound by ~0.3 eV from the experimental benchmark of –1.15 eV[1]. Furthermore, GGA is well-known to overestimate the molecular O_2 bond energy[2]: we obtain 5.65 eV with PBE, overestimated by 0.53 eV from the experimental value of 5.12 eV[3]. To obtain a more realistic gas-phase O_2 reference, we apply an energy correction of $\delta_{\mathrm{O}_2} = +0.53$ eV per O_2 molecule in our thermodynamic analysis. We do not apply the commonly employed correction of 1.36 eV per O_2 molecule proposed by Wang *et al.*[4] for two main reasons. First, the value was extracted from formation enthalpies of nontransition metal oxides, which are chemically different from our metallic ensembles with chemisorbed O. More importantly, because 1.36 eV is much larger than our PBE bond energy error, the correction would over-destabilize O_2 and consequently further overestimate the adsorption energy.

The relative energies are evaluated in terms of the Gibbs free energy of surface segregation, normalized by the number of Pd atoms, with and without adsorbate X = O, H (Supplementary Fig. 5):

$$G_{\mathrm{seg}} = \frac{E\left[x\mathrm{X}_{(\mathrm{ads})}/\mathrm{Pd}_n^{\mathrm{surf}}\mathrm{Au}(111)\right] + (n-1)E[\mathrm{Au}(111)] - nE\left[\mathrm{Pd}_1^{\mathrm{sub}}\mathrm{Au}(111)\right] - \frac{x}{2}G\left[\mathrm{X}_{2(\mathrm{g})}\right]}{n} \quad (2)$$

Here, (n, x) are the number of (Pd, X) atoms in the unit cell. We primarily consider n = 1-3 (up to Pd trimer) and $x = 0, 2$ (without and with chemisorbed O_2, respectively). The energy is referenced to gas-phase X_2 and isolated Pd monomers in the subsurface layer of Au(111), labeled $\mathrm{Pd}_1^{\mathrm{sub}}\mathrm{Au}(111)$, which is the thermodynamically favored configuration in vacuum. As a first approximation, we only consider the translational and rotational entropies of the gas-phase molecule at the experimental pretreatment conditions of O_2 at 0.20 atm and 500 °C; and H_2 at 0.25 atm and 150 °C. The vibrational contributions are typically small for slab systems[5] and hence not considered in this analysis.

Extended surface Pd is also considered as the limiting case of larger ensembles: Pd monolayer with a full coverage of H; and a surface Pd oxide model with stoichiometry Pd_5O_4 from a previous study of Pd/Ag(111)[6]. The oxide unit cell consists of 35 Pd atoms and 28 O atoms (labeled $\mathrm{Pd}_{35}\mathrm{O}_{28}$) on top of three layers of 48 Au atoms (labeled $\mathrm{Au}_{3\times48}$). The Brillouin zone is sampled using a Monkhorst-Pack 3×1×1 *k*-point grid[7]. The segregation free energy is defined as:

$$G_{\mathrm{seg}}[\mathrm{Pd}_5\mathrm{O}_4/\mathrm{Au}(111)] = \frac{E[\mathrm{Pd}_{35}\mathrm{O}_{28}/\mathrm{Au}_{3\times48}]+\left(34+\frac{13}{4\times48}\right)E[\mathrm{Au}_{4\times48}]-35E[\mathrm{Pd}_1^{\mathrm{sub}}\mathrm{Au}_{(4\times48)-1}]-14G[\mathrm{O}_{2(\mathrm{g})}]}{35} \quad (3)$$

Here, the energy is referenced to gas-phase O_2 and four layers of 48 Au atoms with an isolated Pd monomer in the subsurface layer (labeled $\mathrm{Pd}_1^{\mathrm{sub}}\mathrm{Au}_{(4\times48)-1}$). The energy of $\mathrm{Au}_{4\times48}$ per atom is used to balance the difference of 13 Au atoms upon formation of the oxide layer with 35 Pd atoms.

2. DFT calculation: H spillover on Pd/Au(111)

HD exchange involves three classes of elementary steps (Supplementary Tables 4-6): (i) dissociative adsorption of H_2 and D_2; (ii) spillover and migration of atomic H or D across different active sites (so-called "scrambling"); and (iii) recombinative desorption of HD. On dilute Pd ensembles, it is energetically favorable for chemisorption of H_2 and D_2 to occur on separate

ensembles to minimize lateral repulsion. Consequently, the resulting H/D atoms must undergo spillover from Pd to the Au surface and migrate to a different ensemble for HD formation to occur. In most cases, atomic H/D chemisorption on Pd_n site (n = 1-3) results in two types of species: one coordinated to n Pd atoms (denoted $H_1^{(n)}$ and $D_1^{(n)}$); and the other coordinated to only 1 Pd atom at the ensemble boundary (denoted $H_1^{(1)}$ and $D_1^{(1)}$). Across all of our models, spillover exhibits a rather consistent energy barrier of 0.2-0.3 eV per Pd-H/D bond broken. As such, it is $H_1^{(1)}/D_1^{(1)}$, rather than $H_1^{(n)}/D_1^{(n)}$, that preferentially undergoes spillover. The subsequent migration on Au is facile, with barriers of only 0.05 and 0.19 eV on terrace and step edges, respectively (Supplementary Fig. 6a). We note that the tunneling effect, which would require a full quantum mechanical treatment of the H/D nuclei, remains negligible at reaction temperatures of interest (323 K and above)[8]. Taken together, spillover and migration remain of secondary importance compared to chemisorption/desorption in terms of the overall reaction kinetics.

3. Microkinetic modeling: Reaction mechanisms and energetics

Temperature programmed desorption experiments have established that H and D atoms do not adsorb as strongly on dilute Pd/Au(111) as on pure Pd(111)[9,10]. Previous DFT calculations (using the PBE functional with dDsC dispersion corrections) have found H_2 chemisorption on Pd monomers to be endergonic at 90 ℃ under 0.2 bar of H_2[11]. We model H/D exchange over Pd_n/Au(111) ($n = 1, 2, 3$) as a three-fold process. (i) A H_2 molecule adsorbs on the Pd ensemble and dissociates to generate two H ligands. The H atoms can then spill over and exchange across the Au substrate. (ii) A D_2 molecule undergoes the same process. (iii) The resulting H and D atoms combine on one of the Pd ensembles to form and desorb as a HD molecule.

On Pd_1, H_2 first undergoes a molecular adsorption which is endergonic by +0.23 eV (Supplementary Table 4, reaction 1). By crossing a barrier of 0.37 eV, the H_2 molecule dissociates to generate two H atoms bound to Pd_1 (labeled $Pd_1H_1^{(1)}$; superscript on H indicates H-Pd coordination number), which is endergonic by +0.07 eV relative to the molecularly adsorbed state (Supplementary Table 4, reaction 2). The ensuing spillover from Pd_1 to Au is modeled sequentially. The first H atom must cross a barrier of 0.22 eV to diffuse onto Au, with a similar barrier of 0.23 eV for the second H atom (Supplementary Table 4, reactions 3-4). The same four reactions apply to D_2 as well, with less frequent collision and a more endergonic chemisorption than H_2 due to the larger mass and entropy (Supplementary Table 4, reactions 5-8). Finally, an HD molecule is formed and desorbed after exchanging either a D or H atom across the Au substrate to $Pd_1H_1^{(1)}$ or $Pd_1D_1^{(1)}$ (Supplementary Table 4, reactions 9-12).

The reaction pathways on Pd_2 follows the same three-fold process as on Pd_1. The key difference is that H and D can bind to either one or two Pd atoms. First, H_2 undergoes molecular adsorption which is endergonic by +0.20 eV (Supplementary Table 5, reaction 1), followed by dissociation into two H atoms after crossing a barrier of 0.17 eV (Supplementary Table 5, reaction 2). One H atom binds to two Pd atoms (labeled $H_1^{(2)}$), while the other one binds to only one Pd atom (labeled $H_1^{(1)}$). In contrast to the Pd_1 site, the dissociation on Pd_2 is exergonic by –0.10 eV relative to the molecularly adsorbed state. The adsorption strength of H is proportional to its coordination to Pd, resulting in two different spillover barriers of 0.24 and 0.44 eV for $H_1^{(1)}$ and $H_1^{(2)}$, respectively (Supplementary Table 5, reactions 3-4). Similar energetics are observed for D_2 dissociation (Supplementary Table 5, reactions 5-8). Finally, the HD product molecule is formed by reacting $Pd_2H_1^{(2)}$ or $Pd_2D_1^{(2)}$ with an exchanged H or D atom (Supplementary Table 5, reactions 9-12).

Similar reaction mechanisms are considered on Pd_3. First, H_2 undergoes molecular adsorption which is endergonic by +0.14 eV (Supplementary Table 6, reaction 1), followed by dissociation into two H atoms after crossing a barrier of 0.03 eV (Supplementary Table 6, reaction 2). One H atom binds to three Pd atoms (labeled $H_1^{(3)}$), while the other binds to only one Pd atom (labeled $H_1^{(1)}$). The dissociation is exergonic by –0.36 eV relative to the molecularly adsorbed state. Similar to Pd_2, the spillover barrier of atomic H is proportional to its coordination to Pd, with values of 0.31 and 0.74 eV for $H_1^{(1)}$ and $H_1^{(3)}$, respectively (Supplementary Table 6, reactions 3-4).

4. Microkinetic modeling: Catalytic activity and analysis

At 50 °C, the H/D exchange reaction proceeds slowly on the Pd_1 ensemble. The weak H_2 adsorption on Pd_1 results in a low apparent activation energy of 0.25 eV at 50 °C (Fig. 4a). H_2 chemisorption is also endergonic on Pd_1 by +0.31 eV, compared to being barrierless on Pd(111)[12]. As such, Pd_1 remains bare under reaction conditions of interest. Calculation of the degree of rate control shows that bare Pd_1 is the rate-controlling intermediate, and the main rate-controlling transition states correspond to D_2 dissociation and HD formation (Supplementary Fig. 9a-c). H_2 dissociation and the diffusion of atomic H and D have minor rate-controlling behavior. As such, the apparent activation energy (0.25 eV) is approximately the relative enthalpy of the dissociation transition states (0.22 eV).

Compared to the Pd_1 site, the reaction rate for H/D exchange is much higher on Pd_2 at 50 °C (Supplementary Fig. 4b). Although the apparent activation energy remains low at 0.22 eV, the rate-controlling states are very different (Supplementary Fig. 9d-f). At 50 °C, the Pd_2 sites are partially covered with H and D; as such, $Pd_2H_1^{(2)}$ and $Pd_2D_1^{(2)}$ are the main rate-controlling

intermediates. D_2 dissociation and HD formation correspond to the main rate-controlling transition states. As temperature increases to 150 ℃, surface H and D become scarce, and bare Pd_2 becomes the rate-controlling intermediate, thereby decreasing the apparent activation energy (Fig. 4b). The first D diffusion from $\mathrm{Pd_2D_1^{(2)}D_1^{(1)}}$ also becomes moderately rate-limiting.

Compared to the Pd_1 and Pd_2 sites, H_2 adsorption is the strongest on Pd_3, and the coverages of $\mathrm{H_1^{(3)}}$ and $\mathrm{D_1^{(3)}}$ do not falter in the temperature range of 50-150 ℃. As such, the main rate-controlling intermediates are $\mathrm{Pd_3H_1^{(3)}}$ and $\mathrm{Pd_3D_1^{(3)}}$. On the other hand, D_2 adsorption and HD desorption correspond to the main rate-controlling transition states (Supplementary Fig. 9g-i). D_2 dissociation and HD formation do not exhibit rate-controlling behavior until above 100 ℃. As a result, the apparent activation energy (0.52 eV) (Fig. 4c) is approximately the negative of the adsorption energy of a single H at the 3-fold Pd hollow site in Pd_3 (0.59 eV).

Supplementary Tables

Supplementary Table 1

The best fit coordination numbers (C), bond distances (R), disorder factor (σ^2), energy shifts (ΔE_0), and the NN-XANES derived coordination numbers (C^x).

State	Bond	C	C^x	R (Å)	σ^2(Å^2)	$\Delta E_0(eV)$
S0	Pd-Au	11.1 ± 1.7	10.80 ± 0.02	2.81 ± 0.01	0.007 ± 0.001	-4.6 ± 0.9
	Pd-Pd	0.3 ± 0.6	0.67 ± 0.03	2.77 ± 0.05	0 ± 0.008	
S1	Pd-Au	9.7 ± 1.5	10.56 ± 0.02	2.87 ± 0.01	0.005 ± 0.001	-4.0 ± 0.9
	Pd-Pd	0.8 ± 0.6	0.73 ± 0.01	2.79 ± 0.04	0 ± 0.004	
S2	Pd-Au	12.5 ± 0.5	11.40 ± 0.03	2.80 ± 0.02	0.007 ± 0.004	-4.6 ± 0.2
	Pd-Pd	N/A	0.24 ± 0.05	N/A	N/A	N/A
S3	Pd-Au	12.6 ± 0.7	11.64 ± 0.05	2.80 ± 0.02	0.007 ± 0.004	-4.7 ± 0.4
	Pd-Pd	N/A	0.05 ± 0.05	N/A	N/A	N/A

Supplementary Table 2

Computational set-up of close-packed slab models considered for DFT modeling.

Facet	Features	Unit cell	Number of layers	*k*-point grid
(111)	Close-packed terrace	3 × 3	6 (bottom 3 fixed)	7 × 7 × 1
(211)	A-step edge	3 × 4	4 (bottom 1 fixed)	8 × 5 × 1
(331)	B-step edge	6 × 4	4 (bottom 1 fixed)	4 × 5 × 1

Supplementary Table 3

Statistical thermodynamics expressions of the translational, rotational, and vibrational components of the integrated constant-volume heat capacity ($\int_0^T c_V dT$) and the entropy (S) employed for free energy conversions[13]. m = molecular mass; I = principal moment of inertia; σ = symmetry number ($\sigma = 1$ for HD; $\sigma = 2$ for H_2 and D_2).

Component	$\int_0^T c_V dT$	S
Translational (3D)	$\frac{3}{2} k_B T$	$k_B \left\{ \ln \left[\left(\frac{2\pi m k_B T}{h^2} \right)^{\frac{3}{2}} \frac{k_B T}{P} \right] + \frac{5}{2} \right\}$
Rotational (linear)	$k_B T$	$k_B \left\{ \ln \left[\frac{8\pi^2 I k_B T}{\sigma h^2} \right] + \frac{3}{2} \right\}$
Vibrational (harmonic)	$\sum_i \frac{h\nu_i}{e^{h\nu_i/k_B T} - 1}$	$k_B \sum_i \left[\frac{h\nu_i/k_B T}{e^{h\nu_i/k_B T} - 1} - \ln\left(1 - e^{-h\nu_i/k_B T}\right) \right]$

Supplementary Table 4

The elementary steps of HD exchange reaction on Pd_1/Au(111). The Gibbs free energies at standard pressure and the rate constants are computed at 50 ℃ and 1 bar. Superscript on chemical species indicates its coordination number to Pd.

#	Elementary step	ΔG° (eV)	$\Delta G^{o\ddagger}_{\mathrm{fwd}}$ (eV)	k^{fwd} (s^{-1})	k^{rev} (s^{-1})
1	$\mathrm{Pd_1 + H_{2(g)} \rightleftharpoons H_2Pd_1}$	0.23	N/A	7.65×10^{8}	3.26×10^{12}
2	$\mathrm{H_2Pd_1 \rightleftharpoons Pd_1H_2^{(1)}}$	0.07	0.37	1.04×10^{7}	1.48×10^{8}
3	$\mathrm{Pd_1H_2^{(1)} + Au_1 \rightleftharpoons Pd_1H_1^{(1)} + Au_1H_1}$	0.20	0.22	2.27×10^{9}	2.63×10^{12}
4	$\mathrm{Pd_1H_1^{(1)} + Au_1 \rightleftharpoons Pd_1 + Au_1H_1}$	0.18	0.23	1.87×10^{9}	1.35×10^{12}
5	$\mathrm{Pd_1 + D_{2(g)} \rightleftharpoons D_2Pd_1}$	0.24	N/A	5.41×10^{8}	3.40×10^{12}
6	$\mathrm{D_2Pd_1 \rightleftharpoons Pd_1D_2^{(1)}}$	0.10	0.39	4.71×10^{6}	1.46×10^{8}
7	$\mathrm{Pd_1D_2^{(1)} + Au_1 \rightleftharpoons Pd_1D_1^{(1)} + Au_1D_1}$	0.20	0.23	1.54×10^{9}	2.08×10^{12}
8	$\mathrm{Pd_1D_1^{(1)} + Au_1 \rightleftharpoons Pd_1 + Au_1D_1}$	0.19	0.24	1.32×10^{9}	1.07×10^{12}
9	$\mathrm{Pd_1 + HD_{(g)} \rightleftharpoons HDPd_1}$	0.26	N/A	6.25×10^{8}	6.49×10^{12}
10	$\mathrm{HDPd_1 \rightleftharpoons Pd_1H_1^{(1)}D_1^{(1)}}$	0.08	0.38	8.56×10^{6}	1.63×10^{8}
11	$\mathrm{Pd_1H_1^{(1)}D_1^{(1)} + Au_1 \rightleftharpoons Pd_1H_1^{(1)} + Au_1D_1}$	0.20	0.23	1.57×10^{9}	2.08×10^{12}
12	$\mathrm{Pd_1H_1^{(1)}D_1^{(1)} + Au_1 \rightleftharpoons Pd_1D_1^{(1)} + Au_1H_1}$	0.20	0.22	2.22×10^{9}	2.63×10^{12}

Supplementary Table 5

The elementary steps of HD exchange reaction on Pd_2/Au(111). The Gibbs free energies at standard pressure and the rate constants are computed at 50 ℃ and 1 bar. Superscript on chemical species indicates its coordination number to Pd.

#	Elementary step	$\Delta G°$ (eV)	$\Delta G^{o\ddagger}_{\mathrm{fwd}}$ (eV)	k^{fwd} (s^{-1})	k^{rev} (s^{-1})
1	$\mathrm{Pd_2 + H_{2(g)} \rightleftharpoons H_2Pd_2}$	0.20	N/A	1.53×10^{9}	1.87×10^{12}
2	$\mathrm{H_2Pd_2 \rightleftharpoons Pd_2H_1^{(2)}H_1^{(1)}}$	–0.10	0.17	1.26×10^{10}	4.02×10^{8}
3	$\mathrm{Pd_2H_1^{(2)}H_1^{(1)} + Au_1 \rightleftharpoons Pd_2H_1^{(2)} + Au_1H_1}$	0.17	0.24	1.25×10^{9}	5.51×10^{11}
4	$\mathrm{Pd_2H_1^{(2)} + Au_1 \rightleftharpoons Pd_2 + Au_1H_1}$	0.40	0.44	1.10×10^{6}	1.88×10^{12}
5	$\mathrm{Pd_2 + D_{2(g)} \rightleftharpoons D_2Pd_2}$	0.21	N/A	1.08×10^{9}	1.96×10^{12}
6	$\mathrm{D_2Pd_2 \rightleftharpoons Pd_2D_1^{(2)}D_1^{(1)}}$	–0.08	0.19	6.58×10^{9}	4.21×10^{8}
7	$\mathrm{Pd_2D_1^{(2)}D_1^{(1)} + Au_1 \rightleftharpoons Pd_2D_1^{(2)} + Au_1D_1}$	0.17	0.25	8.77×10^{8}	4.51×10^{11}
8	$\mathrm{Pd_2D_1^{(2)} + Au_1 \rightleftharpoons Pd_2 + Au_1D_1}$	0.41	0.45	7.46×10^{5}	1.60×10^{12}
9	$\mathrm{Pd_2 + HD_{(g)} \rightleftharpoons HDPd_2}$	0.22	N/A	1.25×10^{9}	3.57×10^{12}
10	$\mathrm{HDPd_2 \rightleftharpoons Pd_2H_1^{(2)}D_1^{(1)}}$	–0.09	0.18	9.01×10^{9}	4.07×10^{8}
11	$\mathrm{Pd_2H_1^{(2)}D_1^{(1)} + Au_1 \rightleftharpoons Pd_2H_1^{(2)} + Au_1D_1}$	0.17	0.25	8.90×10^{8}	4.51×10^{11}
12	$\mathrm{Pd_2H_1^{(2)}D_1^{(1)} + Au_1 \rightleftharpoons Pd_2D_1^{(2)} + Au_1H_1}$	0.17	0.24	1.36×10^{9}	5.51×10^{11}

Supplementary Table 6

The elementary steps of HD exchange reaction on Pd_3/Au(111). The Gibbs free energies at standard pressure and the rate constants are computed at 50 °C and 1 bar. Superscript on chemical species indicates its coordination number to Pd.

#	Elementary step	$\Delta G°$ (eV)	$\Delta G^{o\ddagger}_{\mathrm{fwd}}$ (eV)	k^{fwd} (s^{-1})	k^{rev} (s^{-1})
1	$\mathrm{Pd_3 + H_{2(g)} \rightleftharpoons H_2Pd_3}$	0.14	N/A	2.30×10^{9}	3.22×10^{11}
2	$\mathrm{H_2Pd_3 \rightleftharpoons Pd_3H_1^{(3)}H_1^{(1)}}$	–0.36	0.03	2.30×10^{12}	4.72×10^{6}
3	$\mathrm{Pd_3H_1^{(3)}H_1^{(1)} + Au_1 \rightleftharpoons Pd_3H_1^{(3)} + Au_1H_1}$	0.14	0.31	1.08×10^{8}	1.88×10^{10}
4	$\mathrm{Pd_3H_1^{(3)} + Au_1 \rightleftharpoons Pd_3 + Au_1H_1}$	0.66	0.74	2.05×10^{1}	3.89×10^{11}
5	$\mathrm{Pd_3 + D_{2(g)} \rightleftharpoons D_2Pd_3}$	0.15	N/A	1.62×10^{9}	3.38×10^{11}
6	$\mathrm{D_2Pd_3 \rightleftharpoons Pd_3D_1^{(3)}D_1^{(1)}}$	–0.35	0.05	9.68×10^{11}	3.66×10^{6}
7	$\mathrm{Pd_3D_1^{(3)}D_1^{(1)} + Au_1 \rightleftharpoons Pd_3D_1^{(3)} + Au_1D_1}$	0.15	0.32	7.94×10^{7}	1.54×10^{10}
8	$\mathrm{Pd_3D_1^{(3)} + Au_1 \rightleftharpoons Pd_3 + Au_1D_1}$	0.67	0.75	1.27×10^{1}	3.18×10^{11}
9	$\mathrm{Pd_3 + HD_{(g)} \rightleftharpoons HDPd_3}$	0.16	N/A	1.88×10^{9}	6.17×10^{11}
10	$\mathrm{HDPd_3 \rightleftharpoons Pd_3H_1^{(3)}D_1^{(1)}}$	–0.37	0.04	1.57×10^{12}	3.19×10^{6}
11	$\mathrm{Pd_3H_1^{(3)}D_1^{(1)} + Au_1 \rightleftharpoons Pd_3H_1^{(3)} + Au_1D_1}$	0.16	0.33	5.59×10^{7}	1.54×10^{10}
12	$\mathrm{Pd_3H_1^{(3)}D_1^{(1)} + Au_1 \rightleftharpoons Pd_3D_1^{(3)} + Au_1H_1}$	0.15	0.31	9.06×10^{7}	1.88×10^{10}

Supplementary Figures

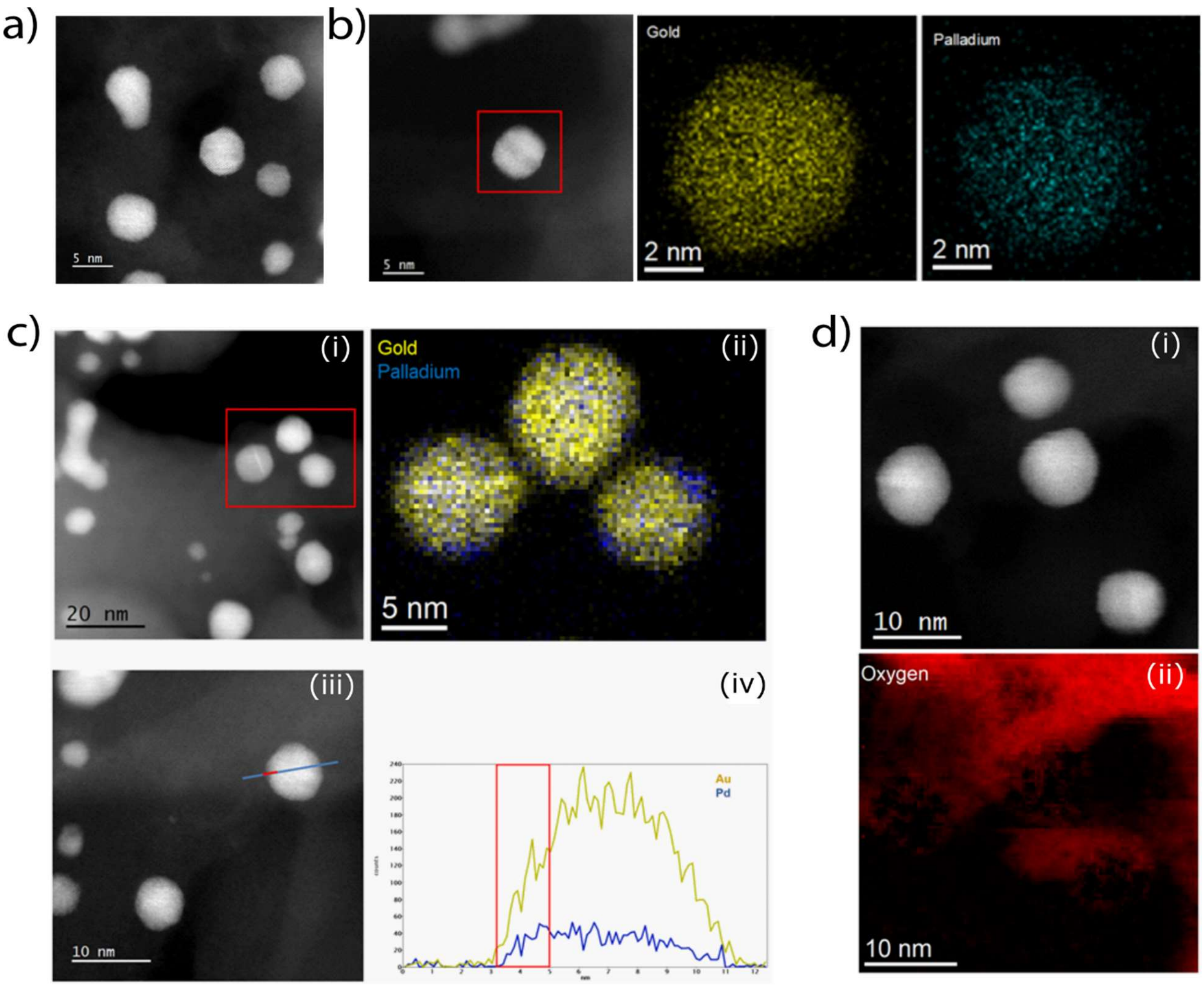

Supplementary Figure 1 | STEM and EDS measurements. (a) Atomic resolution dark-field STEM image of Pd_8Au_{92} particles on RCT-SiO_2. (b) EDS maps of a single particle showing homogeneous elemental distribution, observed in >90% of the particles. (c) Evidence of localized Pd segregation at edges of some particles. EDS map in (ii) corresponds to the red box in (i). Line scan in (iv) corresponds to the cross-section in (iii) with dimmer edge region indicating an excess of Pd. (d) (i) STEM image of four particles and (ii) the EELS map for the O K-edge. The O signal emanates only from the silica support.

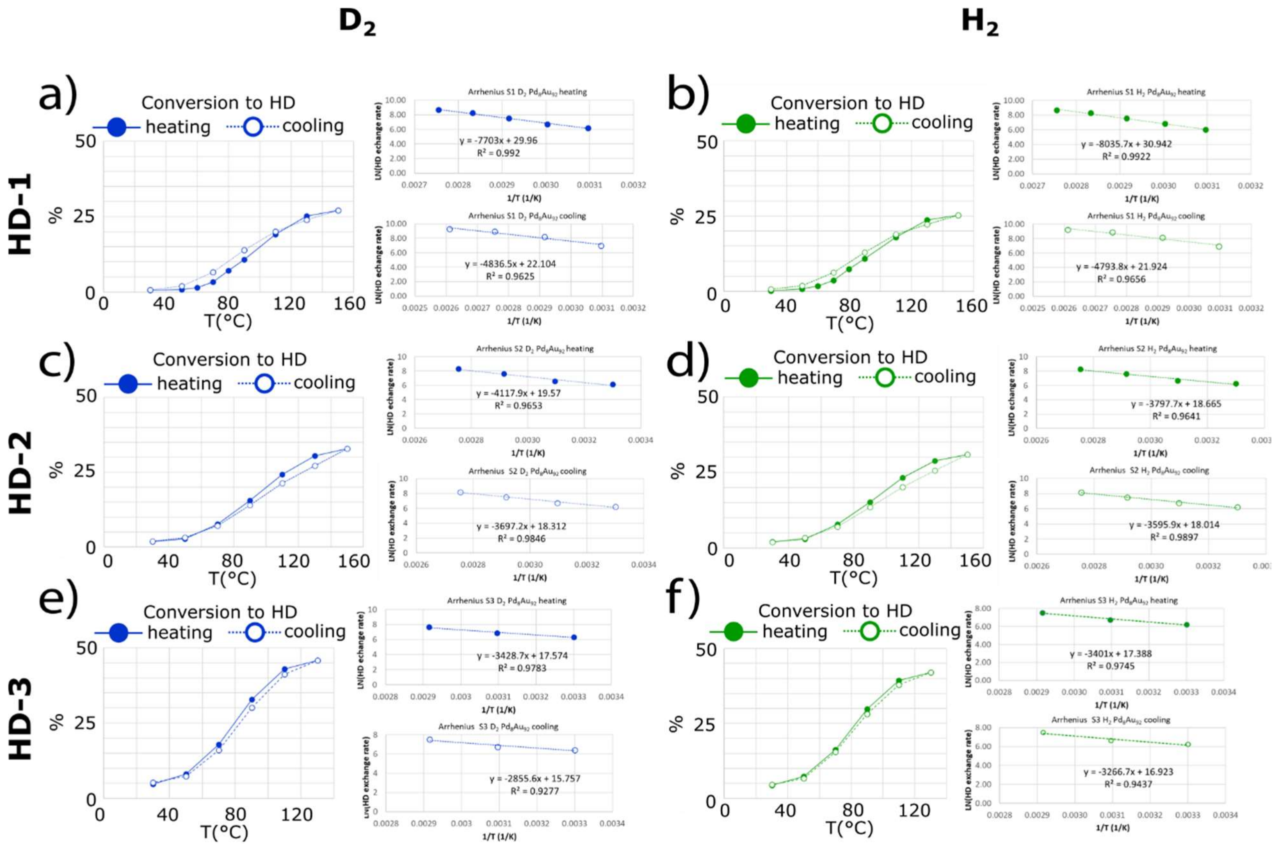

Supplementary Figure 2 | HD exchange reaction data. The conversion and the Arrhenius plots calculated from D_2 (a, c, e) and H_2 (b, d, f) are shown for the experiments HD-1 (sample S1), HD-2 (sample S2), and HD-3 (sample S3) with Pd_8Au_{92}/RCT-SiO_2.

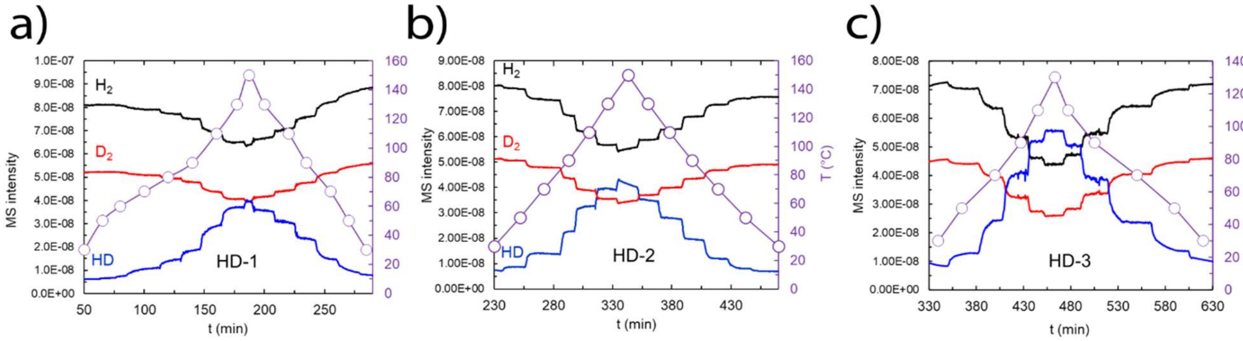

d)

Sample composition	Sample weight (mg)	Treatment (resulting state)	Experiment	E_a (eV) heating	E_a (eV) cooling	*A* heating	*A* cooling
Pd_8Au_{92}	2.1	A (S1)	HD-1	0.67(5)	0.41(5)	30	22
Pd_8Au_{92}	4.8	B (S2)	HD-2	0.34(5)	0.31(5)	18	18
Pd_8Au_{92}	11	C (S3)	HD-3	0.31(5)	0.29(5)	18	18

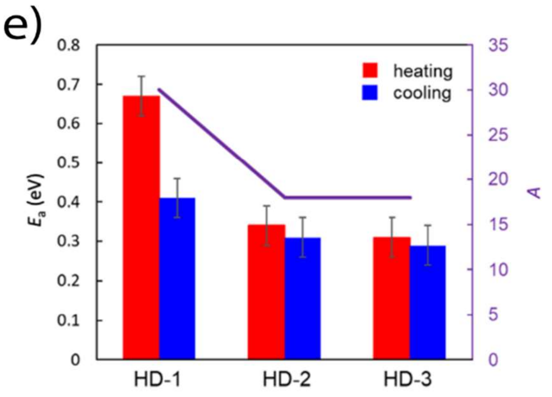

Supplementary Figure 3 | HD exchange reaction profiles and kinetic parameters. (a-c) MS signal during the experiments HD-1 (sample S1), HD-2 (sample S2), and HD-3 (sample S3) with Pd_8Au_{92}/RCT-SiO_2. (d-e) Experimental details, apparent activation energies (E_a), and axis intercepts (*A*) obtained from the Arrhenius plot (i.e., the natural log of the rate of conversion vs. 1/T). Error values were calculated from the average between E_a calculated from H_2 signal and D_2 signal. Errors were presented as 2.5 times estimated standard deviation covering 99% of the population.

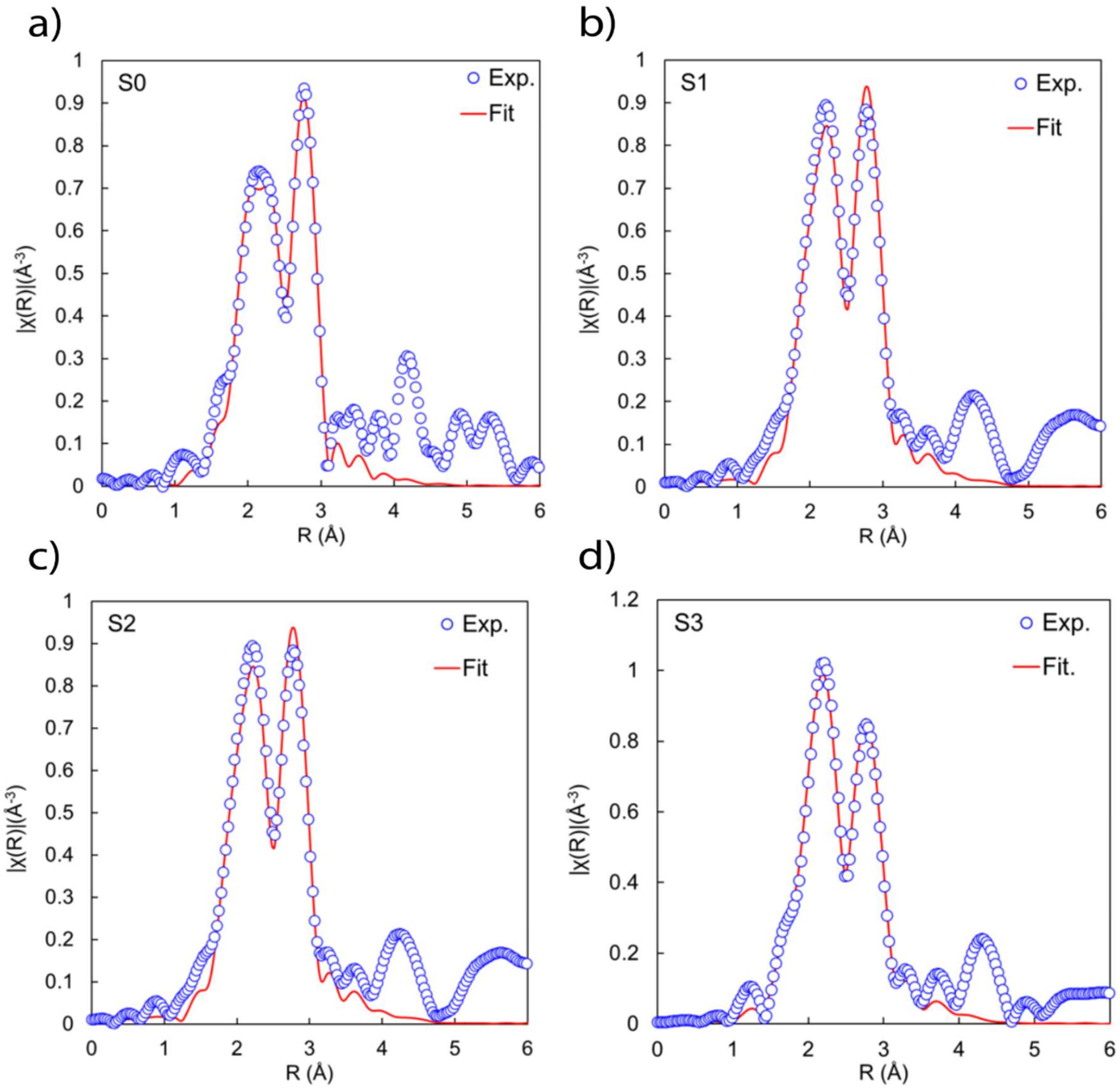

Supplementary Figure 4 | EXAFS data and fits. Comparison between the experimental and fitted EXAFS data in r-space for samples S0 (a), S1 (b), S2 (c) and S3 (d).

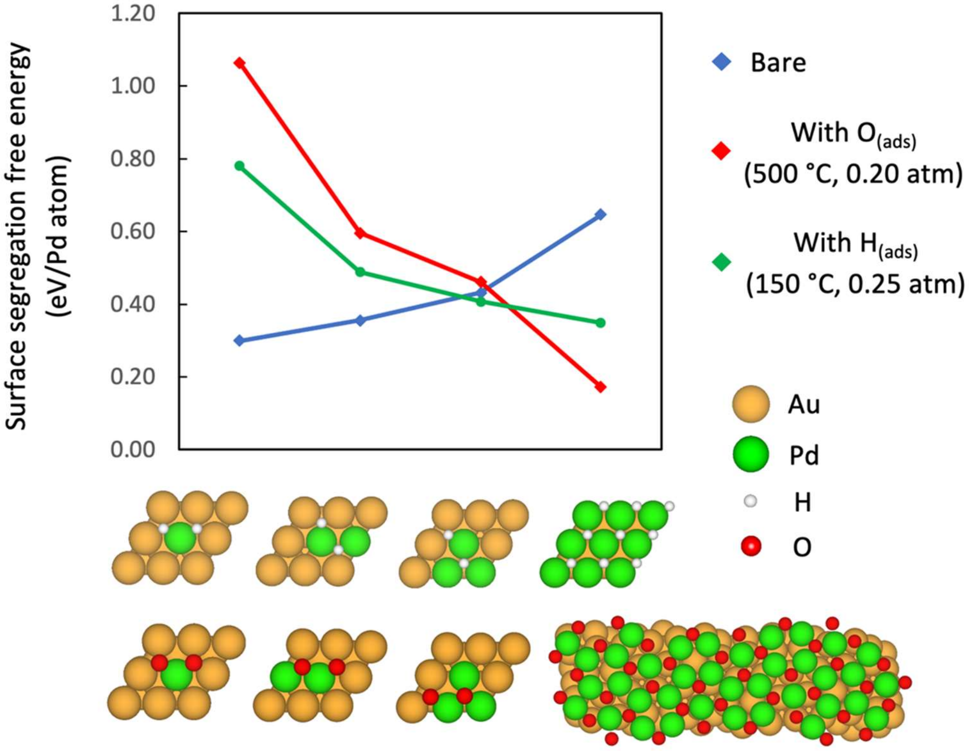

Supplementary Figure 5 | Surface segregation of Pd/Au(111). DFT-computed Gibbs free energies of surface segregation for Pd monomer, dimer, and trimer, as well as extended Pd models (Pd monolayer and surface oxide Pd_5O_4 from a previous study of Pd/Ag(111)[6]), referenced to subsurface Pd monomers and gas-phase O_2 and H_2. A correction of +0.53 eV per O_2 molecule is applied to address the DFT overestimation of the bond energy. The relative stability of the ensembles is inverted upon chemisorption. O_2 provides a strong thermodynamic driving force to form larger metastable ensembles.

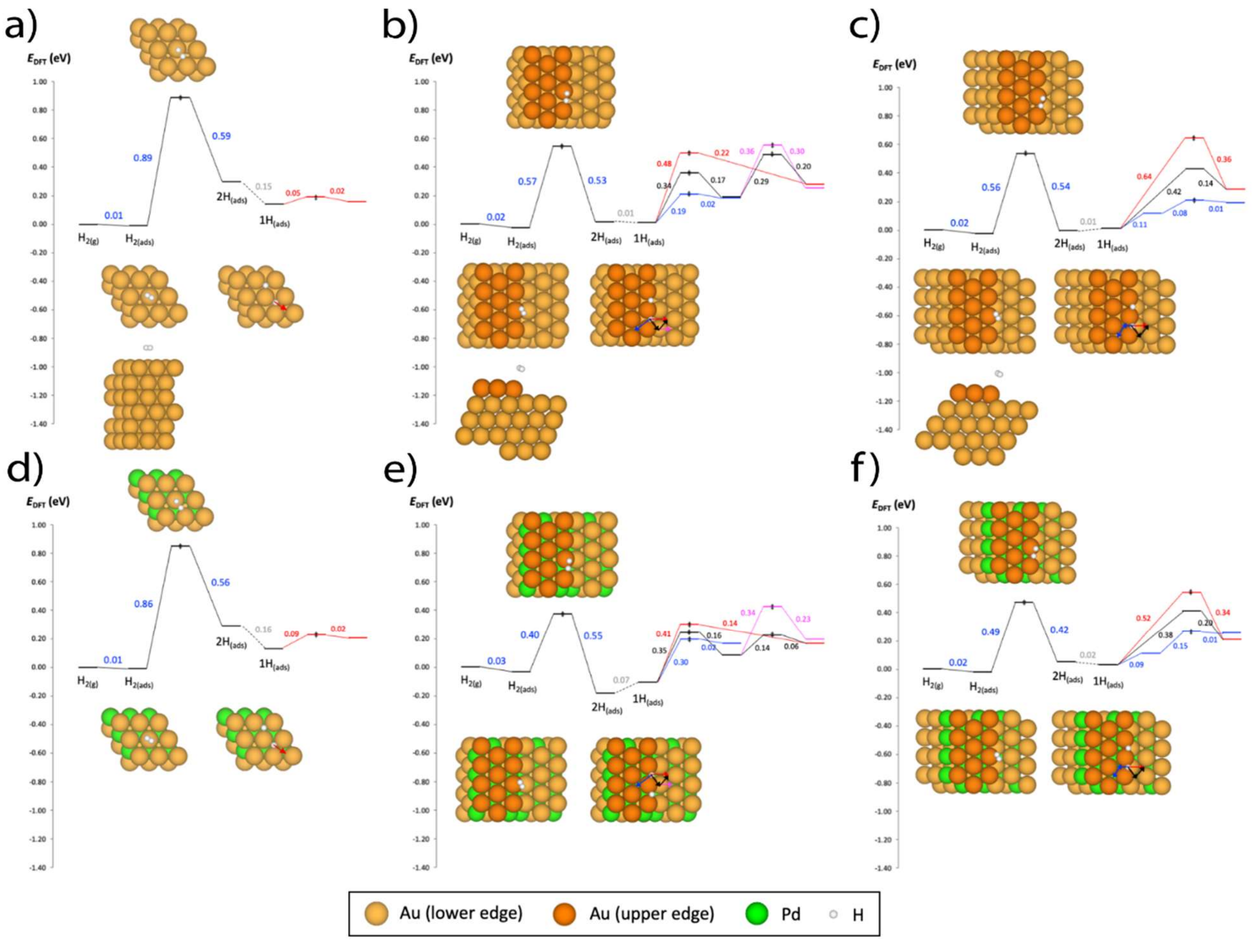

Supplementary Figure 6 | Transition state pathways on extended Au surfaces. DFT-computed potential energy diagram for H_2 dissociative adsorption followed by atomic H diffusion on (a-c) pure Au and (d-f) subsurface Pd monolayer with (111), (211), and (331) facets (left to right). Numbers indicate energy differences between adjacent states. Diffusion pathways are labeled with different colors. Transition states are labeled with ‡. The dotted line indicates a change of reference from $H_{2(g)}$ to ½ $H_{2(g)}$ for $1H_{(ads)}$ states.

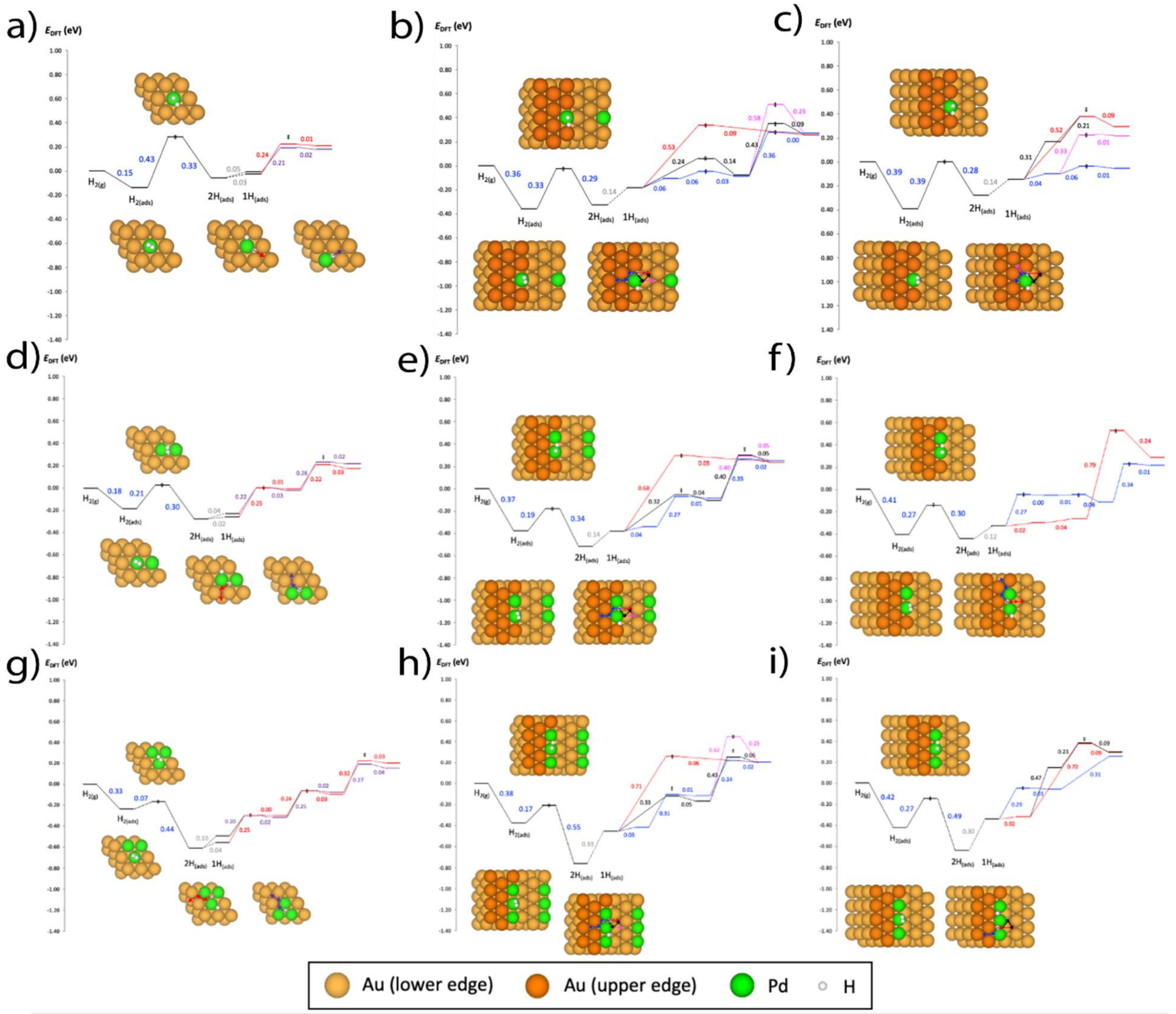

Supplementary Figure 7 | Transition state pathways on dilute Pd ensembles. DFT-computed potential energy diagram for H_2 dissociative adsorption followed by atomic H diffusion on (a-c) Pd monomer, (d-f) dimer, and (g-i) trimer with (111), (211), and (331) facets (left to right). Numbers indicate energy differences between adjacent states. Diffusion pathways are labeled with different colors. Transition states are labeled with ‡. The dotted line indicates a change of reference from $H_{2(g)}$ to ½ $H_{2(g)}$ for $1H_{(ads)}$ states.

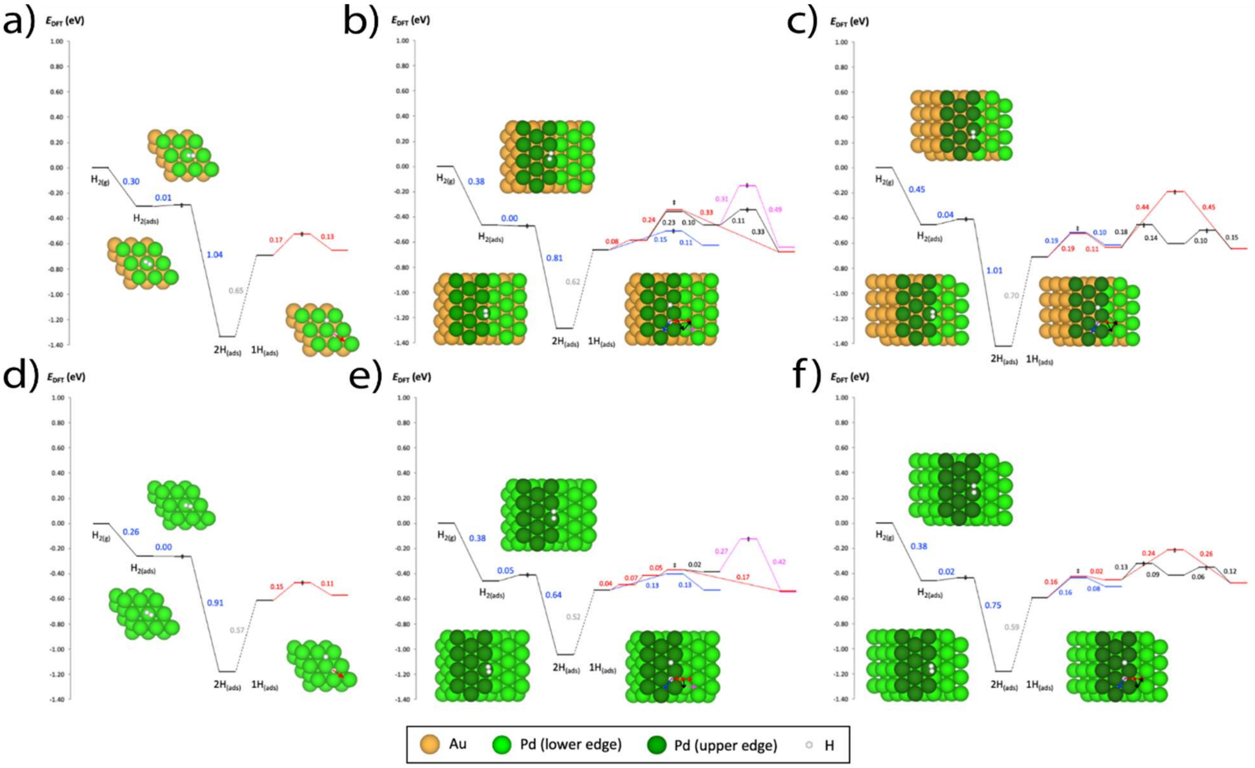

Supplementary Figure 8 | Transition state pathways on extended Pd surfaces. DFT-computed potential energy diagram for H_2 dissociative adsorption followed by atomic H diffusion on (a-c) Pd monolayer and (d-f) pure Pd with (111), (211), and (331) facets (left to right). Numbers indicate energy differences between adjacent states. Diffusion pathways are labeled with different colors. Transition states are labeled with ‡. The dotted line indicates a change of reference from $H_{2(g)}$ to ½ $H_{2(g)}$ for $1H_{(ads)}$ states.

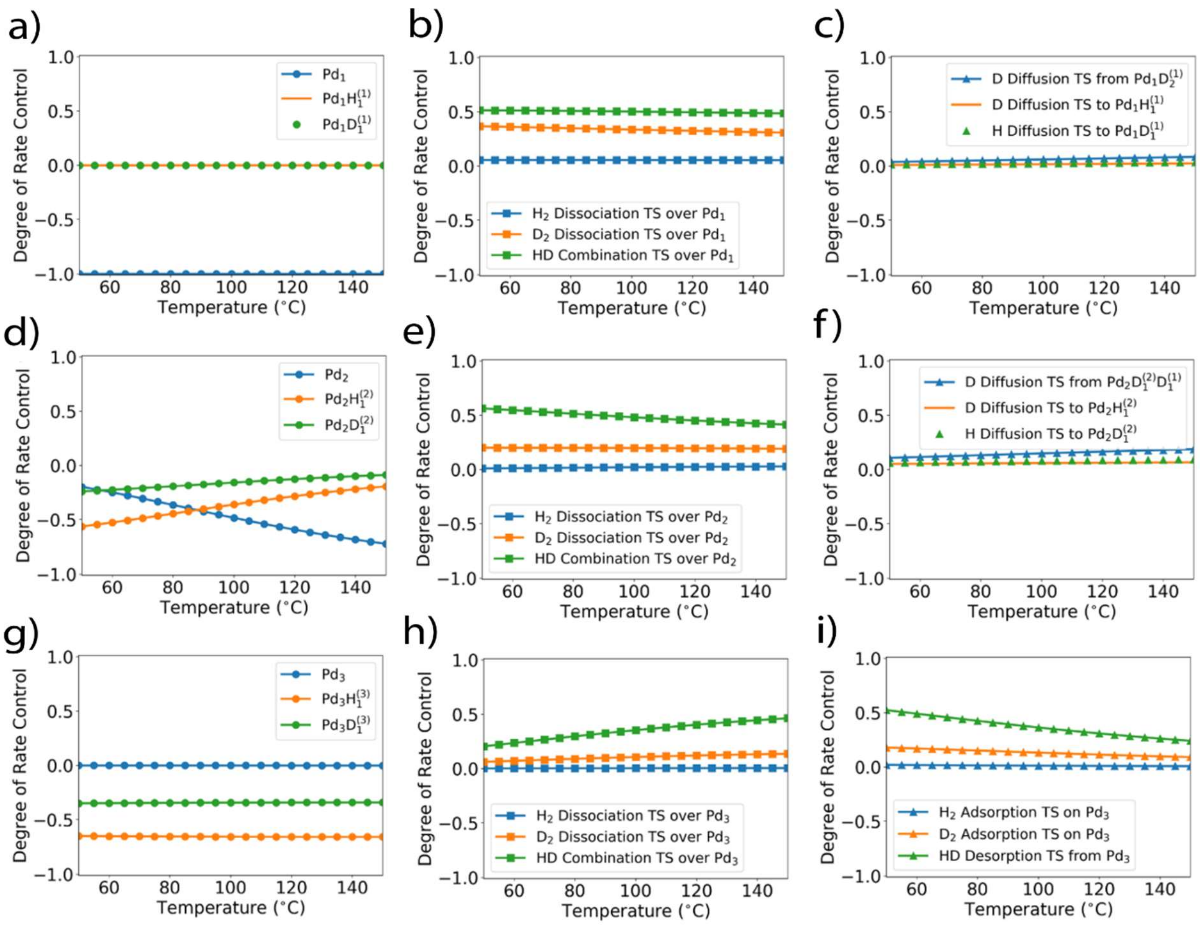

Supplementary Figure 9 | The degrees of rate control for key intermediates and transition states (TS) on dilute Pd ensembles. (a-c) On Pd monomers, the bare site is the rate-controlling intermediate, and the rate-controlling TS corresponds to D_2 dissociation and HD formation. (d-f) On Pd dimers, as the temperature increases, the rate-controlling intermediate switches from $Pd_2H_1^{(2)}$ and $Pd_2D_1^{(2)}$ to bare Pd_2, while the rate-controlling TS corresponds to D_2 dissociation and HD formation. (g-i) On Pd trimers, the rate-controlling intermediates are $Pd_3H_1^{(2)}$ and $Pd_3D_1^{(3)}$. In contrast to monomers and dimers, the rate-controlling TS corresponds to D_2 adsorption and HD desorption at 50 °C.

Supplementary References

1. Guo, X. C., Hoffman, A. & Yates, J. T. Adsorption kinetics and isotopic equilibration of oxygen adsorbed on the Pd(111) surface. J. Chem. Phys. 90, 5787–5792 (1989).

2. Wellendorff, J. et al. A benchmark database for adsorption bond energies to transition metal surfaces and comparison to selected DFT functionals. Surf. Sci. 640, 36–44 (2015).

3. Herzberg, G. Molecular Spectra and Molecular Structure. I. Spectra of Diatomic Molecules. Am. J. Phys. 19, 390–391 (1951).

4. Wang, L., Maxisch, T. & Ceder, G. Oxidation energies of transition metal oxides within the GGA+U framework. Phys. Rev. B 73, 195107 (2006).

5. Reuter, K. & Scheffler, M. Composition, structure, and stability of RuO2 (110) as a function of oxygen pressure. Phys. Rev. B 65, 35406 (2001).

6. van Spronsen, M. A. et al. Dynamics of Surface Alloys: Rearrangement of Pd/Ag(111) Induced by CO and O2. J. Phys. Chem. C 123, 8312–8323 (2019).

7. Monkhorst, H. J. & Pack, J. D. Special points for Brillouin-zone integrations. Phys. Rev. B 13, 5188–5192 (1976).

8. Kristinsdóttir, L. & Skúlason, E. A systematic DFT study of hydrogen diffusion on transition metal surfaces. Surf. Sci. 606, 1400–1404 (2012).

9. Lucci, F. R. et al. Controlling Hydrogen Activation, Spillover, and Desorption with Pd–Au Single-Atom Alloys. J. Phys. Chem. Lett. 7, 480–485 (2016).

10. Yu, W.-Y., Mullen, G. M., Flaherty, D. W. & Mullins, C. B. Selective Hydrogen Production from Formic Acid Decomposition on Pd–Au Bimetallic Surfaces. J. Am. Chem. Soc. 136, 11070–11078 (2014).

11. van der Hoeven, J. E. S. et al. Entropic Control of HD Exchange Rates over Dilute Pd-in-Au Alloy Nanoparticle Catalysts. ACS Catal. 11, 6971–6981 (2021).

12. Busnengo, H. F., Salin, A. & Dong, W. Representation of the 6D potential energy surface for a diatomic molecule near a solid surface. J. Chem. Phys. 112, 7641 (2000).

13. Cramer, C. J. Essentials of Computational Chemistry: Theories and Models. (Wiley, 2004).
